# Trajectories of working memory and decision making abilities along juvenile development in mice

**DOI:** 10.3389/fnins.2025.1524931

**Published:** 2025-02-28

**Authors:** Ann Marlene Thies, Irina Pochinok, Annette Marquardt, Maria Dorofeikova, Ileana L. Hanganu-Opatz, Jastyn A. Pöpplau

**Affiliations:** Institute of Developmental Neurophysiology, Center for Molecular Neurobiology, Hamburg Center of Neuroscience, University Medical Center Hamburg-Eppendorf, Hamburg, Germany

**Keywords:** working memory, decision making, mouse behavior, development, prefrontal cortex, cFos

## Abstract

Rodents commonly serve as model organisms for the investigation of human mental disorders by capitalizing on behavioral commonalities. However, our understanding of the developmental dynamics of complex cognitive abilities in rodents remains incomplete. In this study, we examined spatial working memory as well as odor-and texture-based decision making in mice using a delayed non-match to sample task and a two-choice set-shifting task, respectively. Mice were investigated during different stages of development: pre-juvenile, juvenile, and young adult age. We show that, while working memory abilities in mice improve with age, decision making performance peaks during juvenile age by showing a sex-independent trajectory. Moreover, cFos expression, as a first proxy for neuronal activity, shows distinct age-and brain area-specific changes that relate to task-specific behavioral performance. The distinct developmental trajectories of working memory and decision making in rodents resemble those previously reported for humans.

## 1 Introduction

The term cognition comprises complex abilities that enable an individual to acquire, store, retrieve, and process information in a permanent tradeoff between stability and flexibility of mental representations. These abilities are present in all mammalian species and reach the highest sophistication in humans (Miller and Cohen, 2001). Among them, working memory (WM) and decision making (DM) are of critical relevance. Being the pre-requisite of planning, the WM defines the cognitive capability to temporarily maintain and manipulate information (Baddeley, 1992). Decision making (DM) defines the ability to use a set of cues and previous experience to make a distinct choice to develop a problem-solving strategy. Overall, these abilities belong to the large cognitive flexibility spectrum, which enables an individual to continuously adapt and change acting strategies in response to varying environmental conditions. While many brain areas contribute and interact to enable cognitive flexibility, the prefrontal cortex (PFC) is commonly accepted as a critical hub. The PFC provides “top-down” control by processing incoming information to deliver instructions that suit the behavioral goal (Hanganu-Opatz et al., 2023; Buschman and Miller, 2014).

Impairments in cognitive flexibility, including WM and DM abilities, are key symptoms of neuropsychiatric disorders such as schizophrenia and autism spectrum disorders (Meyer-Lindenberg, 2010). Consequently, the PFC is of central clinical relevance, as it shows major dysfunction in patients, particularly during cognitive tasks (Senkowski and Gallinat, 2015; Goldman-Rakic, 1999). The onset of these disorders typically occurs during development, with early behavioral symptoms often detectable in high-risk prodromal groups (Leicht et al., 2016). The timing of disease onset coincides with the relatively late maturation of cognitive abilities, when compared to sensory and motor functions, and parallels with the delayed structural and functional development of the PFC. Prefrontal developmental milestones follow similar trajectories across mammalian species, including humans and rodents (Klune et al., 2021; Popplau and Hanganu-Opatz, 2024). During adolescence an overproduction of dendritic spines occurs, contributing to a peak in gray matter volume. This is followed by synaptic pruning which results in a strengthening of remaining connections and white matter augmentation (Kolk and Rakic, 2022; Pöpplau et al., 2023; Klein et al., 2014; Petanjek et al., 2011). At the functional level, gamma oscillations, which have been linked to cognitive processing, reach maturity first during adolescence as a product of reciprocal interactions of excitatory and inhibitory neurons (Bitzenhofer et al., 2020; Pöpplau et al., 2023). These structural and functional milestones of the PFC are considered to be critical for the emergence of WM and DM abilities (Popplau and Hanganu-Opatz, 2024; Geier et al., 2009; Crone and Dahl, 2012).

Previous studies have proposed that both in humans and rodents, the developmental dynamic of WM differs from that of DM abilities. In humans, assessments of various WM modalities in children, adolescents, and young adults have shown a steady improvement in WM abilities with age (Heled et al., 2022; Uhlhaas et al., 2009). At the functional level, magnetic resonance imaging data have demonstrated that a better WM performance relates to a more focal activation of prefrontal circuits along development (Geier et al., 2009; Uhlhaas et al., 2009; Kwon et al., 2002; Thomason et al., 2009). In contrast, both juvenile mice and humans have been suggested to exhibit optimal DM performance during the juvenile-adolescent period (Cassotti et al., 2011; Johnson and Wilbrecht, 2011; Hauser et al., 2015; Steinberg, 2004). This period is characterized by novelty-seeking and risk-taking behaviors, which are coupled with a lack of self-regulation. While these traits can lead to poor judgment, they also promote growing independence from caregivers and support the transition to a self-sufficient life (Spear, 2000; Steinberg, 2010; Crone and Dahl, 2012). At the same time, these behaviors make juveniles more flexible and creative in their thinking and problem-solving, which may provide an advantage in DM tasks compared to WM tasks (Cassotti et al., 2011; Crone and Dahl, 2012).

A detailed understanding of the developmental trajectories of WM and DM abilities is crucial for the early identification of behavioral symptoms in neuropsychiatric disorders. Moreover, rodent models that mimic the developmental etiology of these disorders can help to better understand the cellular mechanisms underlying cognitive dysfunction (Chini and Hanganu-Opatz, 2021). For this, it is mandatory to closely align critical developmental events and draw meaningful parallels between species. However, the emergence and developmental dynamics of WM and DM abilities across age in rodents are still poorly understood.

Here, we addressed this knowledge gap by investigating three age groups of C57Bl/6 J mice at pre-juvenile [Pre, postnatal day (P) 20–24], juvenile (Juv, P31-35), and young adult (Adu, P56-60) age and assessed their WM and DM performance using a delayed non-match to sample task and a two-choice attentional set-shifting task, respectively. The age group design was based on previous findings identifying distinct trajectories of prefrontal activity patterns during similar time windows (Pöpplau et al., 2023) as well as on physical markers of puberty and sexual maturity (i.e., Pre, pre-pubertal; Juv, onset of puberty; Adu, sexually mature) (Varlinskaya et al., 2013; Larsen and Luna, 2018; Mayer et al., 2010). The behavioral data were supplemented with immunostaining against cFos in mice which underwent the task as well as in home-cage control mice. We report age-dependent but sex-independent changes in behavioral performance as well as age-and task-specific cFos expression patterns. Overall, our data provide first insights into the functional trajectories of WM and DM abilities in mice.

## 2 Materials and methods

### 2.1 Animals

All experiments were performed in compliance with the German laws and the guidelines of the European Union for the use of animals in research (European Union Directive 2010/63/EU) and were approved by the local ethical committee (N18/015, N19/121).

Timed-pregnant C57Bl/6J mice from the animal facility of the Center for Molecular Neurobiology Hamburg were housed individually in a 12 h light/12 h dark cycle with water and food *ad libitum*. Humidity and temperature were kept constant (40% relative humidity; 22°C). A confirmed vaginal plug in the morning was considered embryonic day 0.5 and day of birth was considered P0. Pups were weaned at P20 and group-housed with nesting material and nibbling wood. Experiments were carried out on both sexes at pre-juvenile (P20-24), juvenile (P31-35) and adult (P56-60) age.

### 2.2 Spontaneous alternations in a Y-maze

Spontaneous alternations (SpA) were performed at the same age for each group (Pre: P20, Juv: P31, Adu: P56). Prior to the delayed non-match to sample (DNMS) task, SpA were accessed in a custom build Y-maze consisting of three identical arms at a 120° angle to each other. Mice were placed in the experiment room 1 h before SpA for habituation. Home cages were changed before habituation and not changed until the end of the DNMS task to prevent unnecessary stress. Between mice, the maze was cleaned with 0.1% acetic acid to neutralize all odors.

For SpA quantification, mice were placed in the start arm A and allowed to freely explore the maze for 10 min. The maze was covered by a Plexiglas plate to prevent mice from jumping out of the maze. Mice were videotaped from above and were tracked offline using the Python-based tracking system ezTrack (Pennington et al., 2019). Monitored parameters included number of entries, number of alternations, time spent in the center of the maze, direction of alternations, and distance moved for each frame. Percentage of alternations was quantified by dividing the number of alternations by the number of entries. Position change of more than 1 cm/s was considered as movement.

### 2.3 Working memory—delayed non-match to sample task

All WM trials of the DNMS task were performed at the same age for each group (Pre: P20-24, Juv: P31-35, Adu: P56-60) during the first half of the light cycle. After SpA, the Y-maze was extended by a center gate and a lick port in each sample arm to present a drop of corn oil as a reward. Mice were familiarized with the corn oil in their home cage, starting 7 days before the experiment. Between mice, the maze was cleaned with 0.1% acetic acid to neutralize all odors. Throughout WM testing, mice had restricted access to food. Specifically, on the evening before the tests, food was completely removed. On the next day, after performing the tests in the morning, mice had again unlimited access to food until the evening. The weight of the mice was monitored on each day during behavioral testing.

For the sample trial, mice were placed in the start arm A, facing the wall, while arms B and C were open. After the mice chose a sample arm, the gate was lowered, and the mice were allowed to collect the reward. Mice were removed from the maze and the gate was lifted. For the test trial, mice were placed back in the maze with a delay of 5–10 s. Test choice was considered correct when mice visited the arm not explored during the sample trial. For an inter-trial interval of 60 s mice were placed in a separate cage containing some home cage bedding. Testing was performed on five consecutive days with 10 consecutive trials each day. Performance relative to chance for each day was quantified as modulation index of the percentage of correct trials vs. 50% chance level for each day according to the following equation: (% correct trials – 50%)/(% correct trials +50%). To quantify the slope of change of the percentage of correct trials as well as of performance time, for each mouse, a linear regression (*robustfit*) was performed in MATLAB and the resulting slope was multiplied with the intercept for normalization.

### 2.4 Decision making - two-choice attentional set-shifting task

All two-choice set-shifting trials were performed at the same age for each group (Pre: P23-24, Juv: P34-35, Adu: P59-60) during the first half of the light cycle according to a published protocol (Johnson and Wilbrecht, 2011) and modified to span for 2 days. Starting 2 days before the first test day, mice were food-restricted similarly to the spatial WM task (i.e., no food overnight with unlimited access to food from noon to evening) and placed in the experiment room to habituate to the environment. Home cages were changed on habituation day and not changed until the end of the experiment to prevent unnecessary stress. The custom-built arena consisted of a rectangle box halfway divided by a Plexiglas plate, creating two separate compartments that were equipped with a petri dish.

On the first day, mice were group-habituated to the arena. The petri dishes contained a small amount of shavings and one treat per mouse. Habituation was terminated after 10 min or when all treats were eaten. Afterward, mice were individually conditioned to dig for treats using a single petri dish holding one treat while the level of shaving was increased in four steps from a third to maximum volume. For compound discrimination (CD) shavings were mixed with 0.1% ground dried spices by volume. Mice had to discriminate between odor 1 [O1 (garlic)] indicating a treat and odor 2 [O2 (black pepper)]. The position of the petri dishes was randomly altered throughout the task. A digging choice was defined as purposefully moving the shavings with either paws or nose. After an incorrect choice, mice were instantly removed from the maze and a new trial was initiated. Refusing to dig for 180 s was counted as an omission and mice were put back to their home cage for 15–30 min before continuing the experiment. The criterion for all tests was reached if eight out of 10 consecutive choices were correct. Following the shaping, 30 trials of overtraining were performed.

During the second day, mice had to achieve three consecutive task phases. During compound discrimination reversal (CDR), O1 and O2 were reversed, making O2 the correct rewarded digging choice. During intra-dimensional set-shift (IDSS) two novel odors were introduced, odor 3 [O3 (rosemary)] and odor 4 [O4 (thyme)]. Next, we added texture as dimension which became relevant to investigate a kind of extra-dimensional set-shifting (EDSS). Two sets of two pots containing green sequins and black paper snippets were alternately mixed with two novel odors [O5 (coriander) or O6 (cumin)] to vary the combination of texture and odor. The position of the petri dishes was randomly altered throughout all tasks. The rewarded odor for IDSS and compound for EDSS were varied between cohorts to prevent bias. Analyzed parameters were number of trials to reach the criterion, median latency to dig for all trials during one of the task stages, and the variance of trial latencies. Between mice, the maze was cleaned with 0.1% acetic acid to neutralize all odors. The weight of the mice was monitored on each day during behavioral testing.

### 2.5 Immunohistochemistry

Following the behavioral experiments mice were perfused within 0.5–1 h to ensure expression of immediate early genes (Aparicio et al., 2022). Control animals were perfused at the same age without undergoing behavioral testing, mainly co-housed littermates were used and control mice underwent the same room adaptions and a similar food deprivation. Mice were anesthetized with an injection of 10 μL ketamine and xylazine solution [12 mg/mL ketamine (aniMedica, Senden, Germany), 1.6 mg/mL xylazine (WDT, Garbsen, Germany) in 0.9% sterile saline] per g body weight and perfused with 4% paraformaldehyde (PFA, Histofix, Carl Roth, Karlsruhe, Germany). Brains were removed and stored at 4°C in PFA for 2 days before the PFA was exchanged with phosphate buffered saline (PBS). Brains were sectioned coronally with a vibratome (Leica Biosystems, Wetzlar, Germany) at 50 μm for immunohistochemistry and were collected into well plates containing 0.1% Natriumazid (NaAc, Sigma-Aldrich, Taufkirchen, Germany) in PBS for storage.

Free-floating slices were three times washed for 5 min with 1x PBS on a shaker to remove remaining PFA and NaAc. Washed slices were permeabilized and blocked with 0.3% Triton-X-100 (Carl Roth, Karlsruhe, Germany) and 5% normal goat serum (NGS, Cell Signaling Technology Europe, Leiden, The Netherlands) in PBS for 2 h on a shaker at room temperature and incubated with the primary monoclonal rat IgG antibody against cFos (1:1000) (Synaptic Systems Cat# 226017, RRID: AB_2864765) in antibody carrier solution [1x PBS, 1% bovines serum albumin (Capricorn Scientific, Ebersdorfergrund, Germany), 0.3% Triton-X-100] at 4°C overnight. Afterward, slices were three times washed for 10 min with 1x PBS on a shaker. The secondary antibody Alexa 546 goat anti-rat (1:1000) (Thermo Fisher Scientific Cat# A-11081, RRID: AB_2534125) in antibody carrier solution was added and incubated for 2 h on a shaker at room temperature. Slices were two times washed for 10 min with PBS and then mounted on slides with Vectashield mounting medium containing DAPI (Biozol, Eching, Germany) and stored at 4°C until imaging.

### 2.6 cFos data acquisition and cell quantification

Single images (2048 × 2048 pixels) were taken with a confocal microscope (Zeiss, Oberkochen, Germany) using a 20x objective with a 568 nm laser for the secondary antibody stained against cFos. This resulted in a pixel size of 0.16 μm^2^, corresponding to images of 319.5 μm^2^. Brain areas were identified according to the mouse reference atlas from Allen brain atlas (Lein et al., 2007). Cells were detected with Cellpose (Pachitariu and Stringer, 2022; Stringer et al., 2021), a deep learning-based cellular segmentation algorithm in Python 3.8. For single cell nuclei detection, the Cellpose model *cyto2* was selected and used for all analyzed images. Parameters were kept equal for all images and results were validated by visual inspection. Data were imported and analyzed in MATLAB R2021a (MathWorks, Natick, MA). Only cell nuclei with a diameter larger than 7.5 μm were considered for analysis to exclude nuclei of glial cells.

### 2.7 Statistical analysis

Statistical analyses were performed in MATLAB and R Statistical Software (Foundation for Statistical Computing, Austria). Non-nested behavioral data were tested formally for normal distribution using the Kolmogorow-Smirnow-Test (*kstest*) in MATLAB. None of our measured data showed a normal distribution. Therefore, non-parametric tests were used to test significant differences (*p < 0.05; **p < 0.01; ***p < 0.001) between age groups. For this, the *multcompare* function for multi-comparison on the non-parametric Kruskal-Wallis test (*kruskalwallis*) with Bonferroni corrected *post hoc* analysis was used in MATLAB. Parameters of WM performance and weight change for each age group were tested for the hypothesis that their median is zero at the 5% significance level using the Wilcoxon signed rank test (*signrank*) in MATLAB. The statistical results, including sample size, chi-squared, and corresponding *p*-values, are summarized in Statistics Table S1. For statistical analysis of sex differences linear mixed-effect (LME) models were used. Parameter estimation was done using the *lmer* function implemented in the *lme4* R package. To test the significance of sex in the model, a likelihood ratio test against a reduced model was performed in which sex as a random effect was removed (*lmerTest* R package, full model: measured parameters ~ age group + (1|sex); reduced model: measured parameters ~ age group). Statistics of the impact of sex are summarized in Statistics Table S2, including sample size, chi-squared, and corresponding *p*-values.

Nested cFos data were analyzed with LME models with interactions. Animal, slice, and sex were included in the models to account for variability contributed by these factors. Models were implemented using the *lmer* function from the *lmerTest* R package. To evaluate whether adding animal, slice, and sex as random effects significantly improved the model fit, a reduced model (without a respective random effect) was compared to the full model (with a respective random effect) using a likelihood ratio test (*anova* function from *lmerTest* R package). To assess the significance of fixed effects and the interaction, we performed Type III Sum of Squares ANOVA using the *anova* function from the *car* R package. Post-hoc Tukey-adjusted pairwise comparisons were conducted using *emmeans* and contrast functions from *emmeans* R package. Predicted values and effects were visualized using *ggplot2* R package.

For line plots data are presented as mean ± SEM. Data of violin plots are presented as median with 25th and 75th percentile. Shaded area represents the probability distribution of the variable.

## 3 Results

### 3.1 Mice show age-dependent explorative behaviors

**Figure 1:**
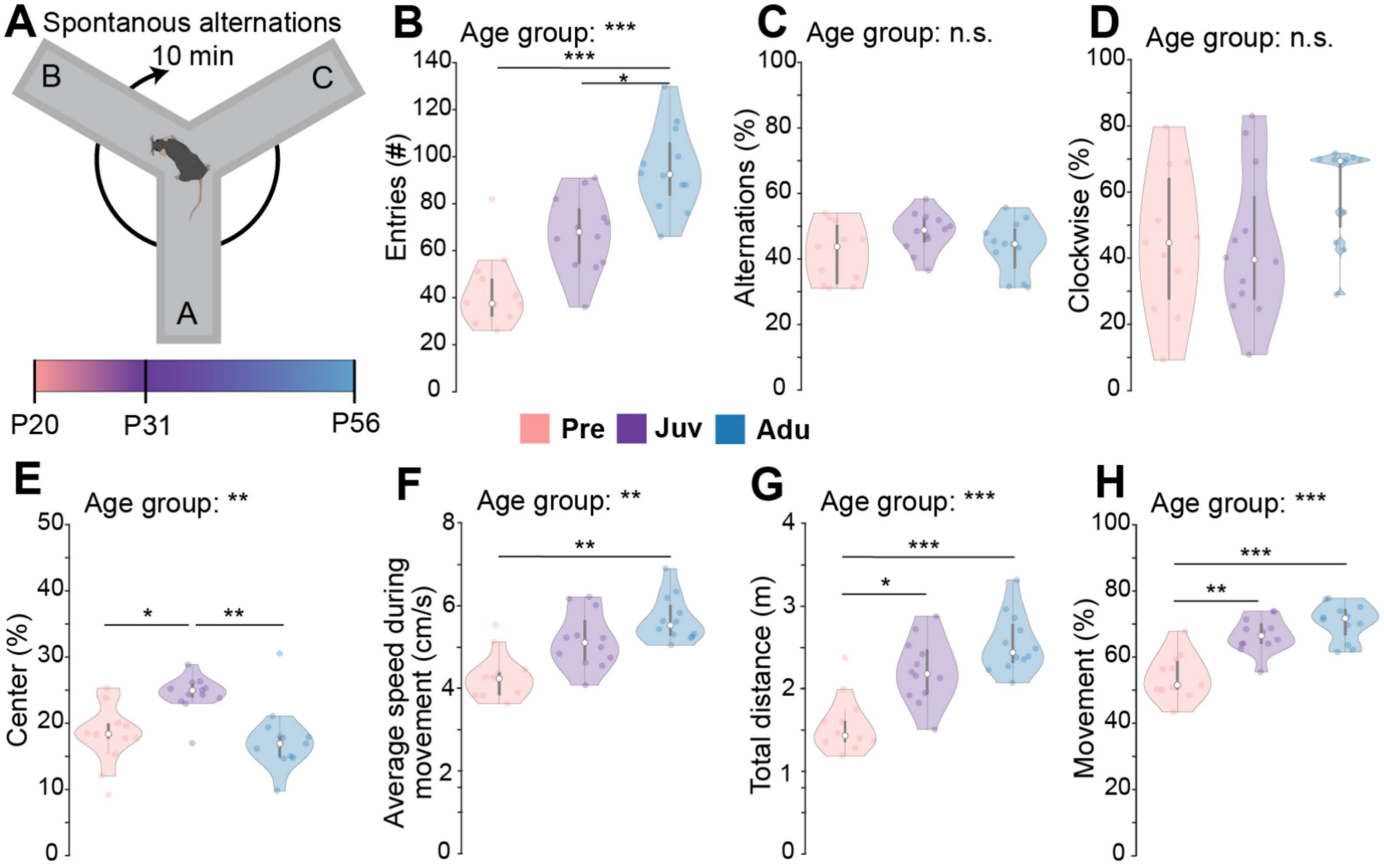
Spontaneous alternations in a Y-maze along mouse development. **(A)** Schematic of the experimental design of the SpA test. **(B)** Violin plots displaying the number of total entries of Pre (*n* = 11), Juv (*n* = 12), and Adu (*n* = 12) mice. **(C)** Same as **(B)** for percentage of alternations. **(D)** Same as **(B)** for percentage of clockwise alternations. **(E)** Same as **(B)** for percentage of time spent in the center of the Y-maze. **(F)** Same as **(B)** for average speed during movement. **(G)** Same as **(B)** for the total distance moved during 10 min of exploration. **(H)** Same as **(B)** for percentage of Movement. Asterisks indicate significant differences between the groups (*p < 0.05, **p < 0.01, ***p < 0.001) and were calculated using the Kruskal-Wallis test with Bonferroni corrected *post hoc*. See also Statistics Table S1 for detailed statistics.

To quantify explorative behavior and innate memory, SpA were assessed in Pre, Juv, and Adu mice (Figure 1A). The number of total entries during 10 min of exploration within a Y-maze increased with age, with Adu mice showing a higher entry count compared to Juv and Pre mice. Nonetheless, the relative number of alternations normalized to the number of entries as well as the direction of alternations showed no difference among the groups (Figures 1B–D). Juv mice spent more time in the center of the maze than the other age groups (Figure 1E). The locomotor activity of Juv and Adu mice was overall similar. However, Pre mice moved slower than Adu mice and generally less than the older mice (Figures 1F–H). Whereas the weight strongly differed between male vs. female mice and generally increased with age for both sexes, mouse locomotor activity as well as SpA were independent of sex (Supplementary Figure S1A and Statistics Table S2). Overall, these results revealed an age-independent ability of mice to navigate through the Y-maze while displaying age-dependent strategies.

### 3.2 Working memory reaches full performance only toward adult age

**Figure 2:**
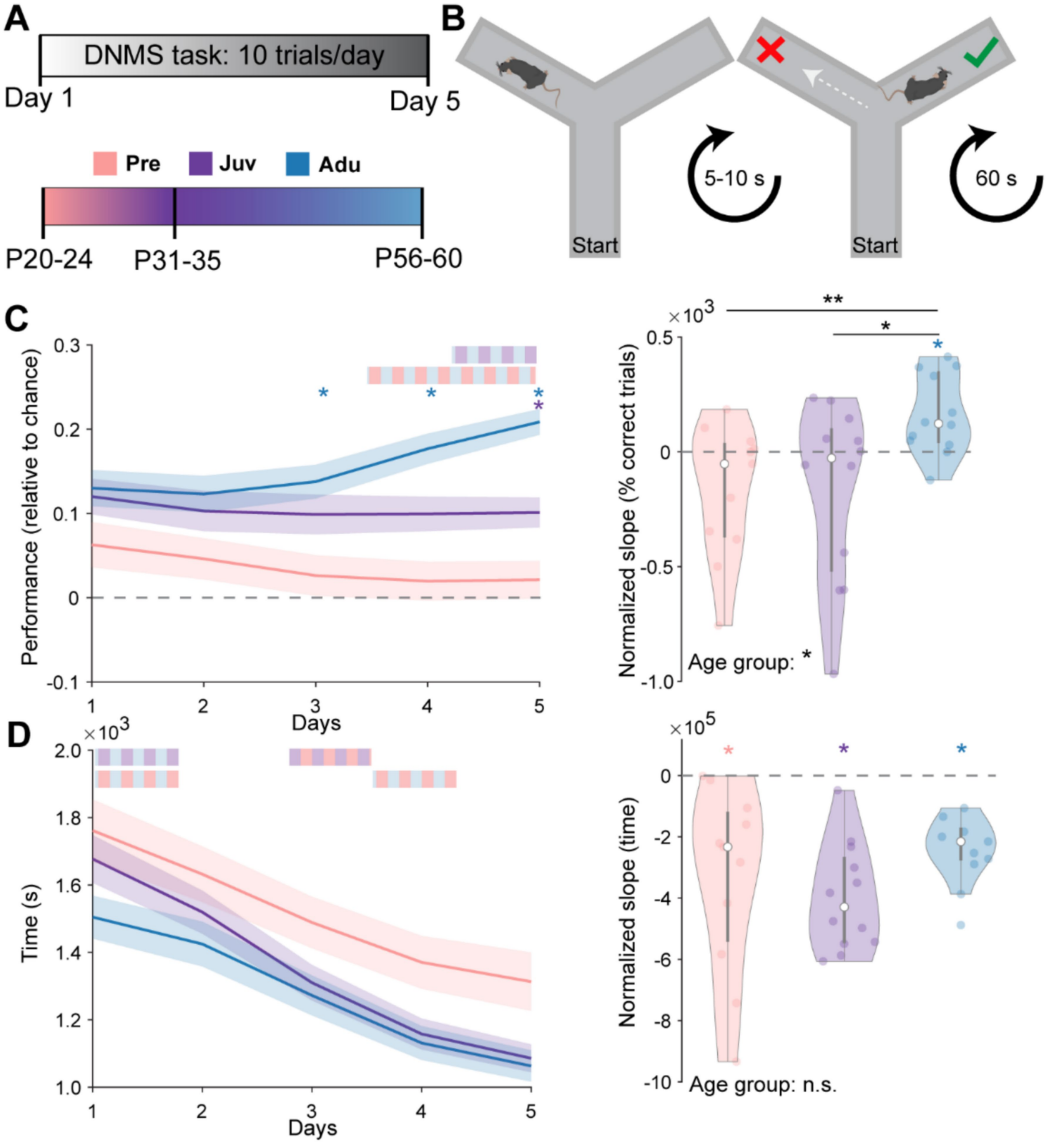
Dynamics of spatial working memory performance along mouse development. **(A)** Schematic showing the experimental timeline and age groups. **(B)** Schematic showing the design of the spatial WM task. **(C)** Left, line plots displaying the WM performance on each day relative to chance level in Pre (*n* = 11), Juv (*n* = 12), and Adu (*n* = 12) mice. Dashed gray line marks the chance level at 50% of correct choices. Right, violin plot displaying the intercept normalized slope of the percentage of correct trials over the 5 days for each mouse of the investigated age groups. **(D)** Same as **(C)** for the average time required on each day to complete a trial (left) as well as the intercept normalized slope of required time over all 5 days (right). Black asterisks and color-striped bars indicate a significant difference between groups (*p < 0.05, **p < 0.01, ***p < 0.001) and were calculated using the Kruskal-Wallis test with Bonferroni corrected post hoc. Colored single stars indicate a significant difference from zero and were calculated using the Wilcoxon signed rank test for zero median. See also Statistics Table S1 for detailed statistics including age group effects for single days in **(C,D)** left.

Subsequent to the SpA, the same mice were tested in the DNMS task during 10 trials per day for a total of five consecutive days (Figures 2A,B). Mice from all three groups were able to terminate all 10 trials on each of the 5 days. However, only Juv on day 5 and Adu mice from day 3 onwards performed above chance level. Whereas Adu mice performed better than Pre mice from day four on, a similar difference to Juv mice was only reached on day five, suggesting a continuous increase in WM abilities with age. Similarly, when quantifying the slope of the percentage of correct trials over the 5 days for each mouse, the median of the Pre and Juv group was not different from zero (i.e., representing no change over days) and showed a significant increase toward adult age. Moreover, the median of Adu mice was above zero, indicating a learning effect at this age (Figure 2C).

All mice performed the 10 trials faster over the 5 days. While Adu mice performed fastest on day one, Juv mice showed the strongest decrease in time to task completion over the 5 days, reaching adult speed on day two. Quantifying the slope of the time change over the 5 days revealed that all mice increased their speed similarly and no group differences could be detected (Figure 2D). All investigated parameters of WM performance were independent of the sex of the mice (Statistics Table S2). Notably, weight changes across days were only moderate and only the weight of Adu mice showed a significant decrease, indicating that the food restriction protocol did not interfere with the developmental increase in weight (Supplementary Figure S1B). Overall, these results demonstrate the linear dynamics of WM development.

### 3.3 Decision making abilities peak at juvenile age

**Figure 3:**
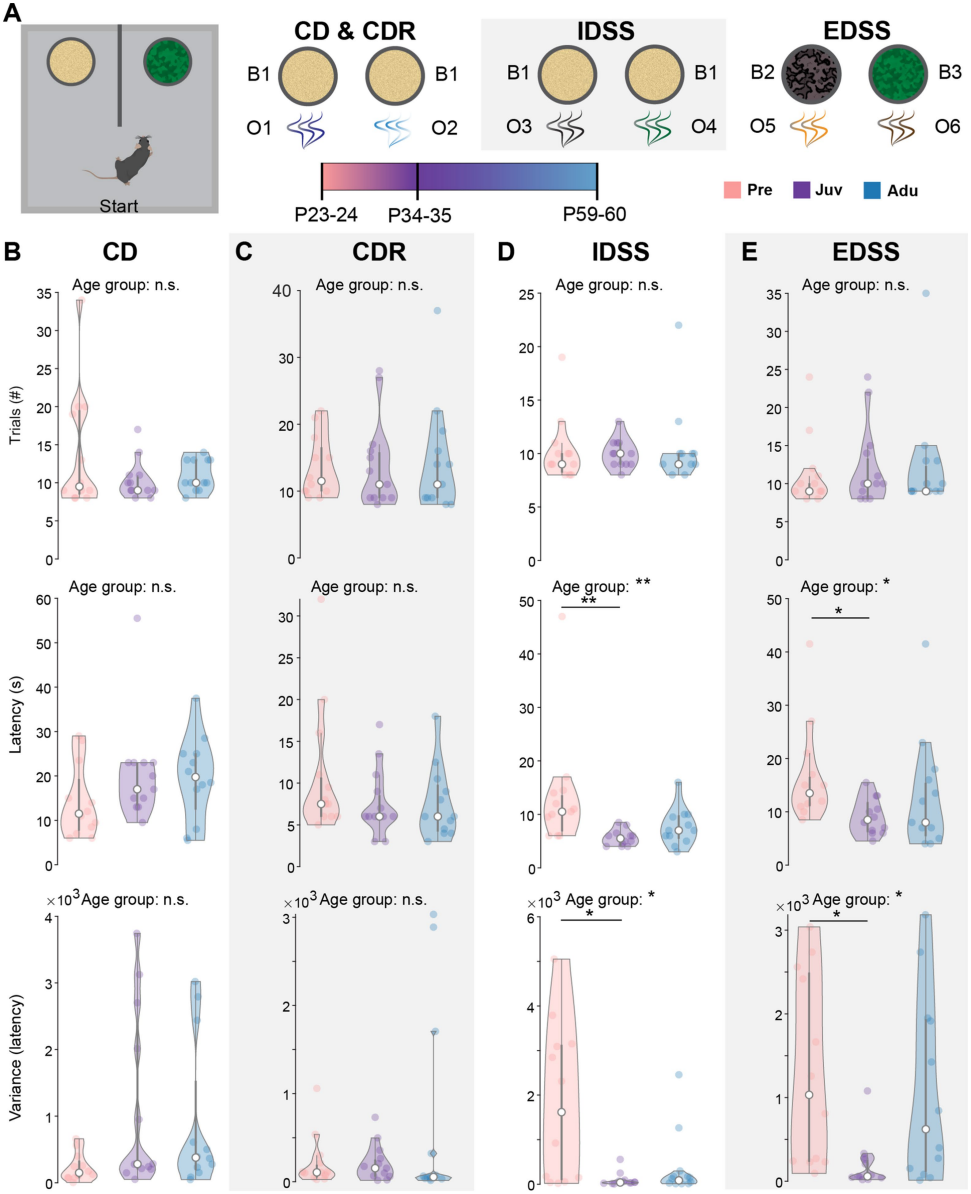
Dynamics of attentional set-shifting abilities along mouse development. **(A)** Schematic showing the experimental design, illustrating the single phases of the two-choice attentional set-shifting task. **(B)** Violin plots showing the number of trials required to reach the criterion (top, i.e., eight out of 10 consecutive choices were correct), the median latency to dig (middle), and the variance of the median latency to dig (bottom) for CD of Pre (*n* = 12), Juv (*n* = 12), and Adu (*n* = 12) mice. **(C)** Same as **(B)** for CDR. **(D)** Same as **(B)** for IDSS. **(E)** Same as **(B)** for EDSS. Asterisks indicate significant differences between the groups (*p < 0.05, **p < 0.01, ***p < 0.001) and were calculated using the Kruskal-Wallis test with Bonferroni corrected post hoc. See also Statistics Table S1 for detailed statistics.

Next, we investigated DM development in a separate cohort of mice that performed a two-choice attentional set-shifting task. Pre, Juv, and Adu mice were tested in their ability to develop, reverse, and switch between different attentional sets based on cues given by odor and texture of digging media (Figure 3A).

During the initial CD learning phase (O1 and O2), mice of all groups showed a similar number of required trials to reach the criterion (i.e., eight out of 10 consecutive choices were correct) and median latency to dig for all trials. Moreover, the temporal variability in their choices was comparable between the groups (Figure 3B). Similar results were achieved for the CDR phase during which the previously not rewarded odor became relevant (Figure 3C). When introducing a new set of odors (O3 and O4) during the IDSS flexibility phase, Pre mice showed a higher latency to dig and larger variability in their choices than Juv mice. No differences were found between Pre and Adu mice (Figure 3D). During the EDSS flexibility phase, the digging media was introduced as a dimension and became relevant instead of odor. Similar to IDSS, during EDSS Juv mice made faster and less variable digging choices than Pre mice, whereas Pre und Adu mice showed a comparable performance (Figure 3E). No differences were found in the required number of trials to reach the criterion for each phase of the task, confirming that all mice are able to learn the task. Notably, similar to WM, DM performance in the age groups was independent of sex (Statistics Table S2). Moreover, protocol-induced weight changes were only moderate and generally similar to WM-tested mice (Supplementary Figures S1C–F). Overall, the better performance of Juv mice compared to Pre mice during IDSS and EDSS suggests a non-linear dynamic of DM abilities along late development.

### 3.4 Basal cFos expression is highest at pre-juvenile age

**Figure 4:**
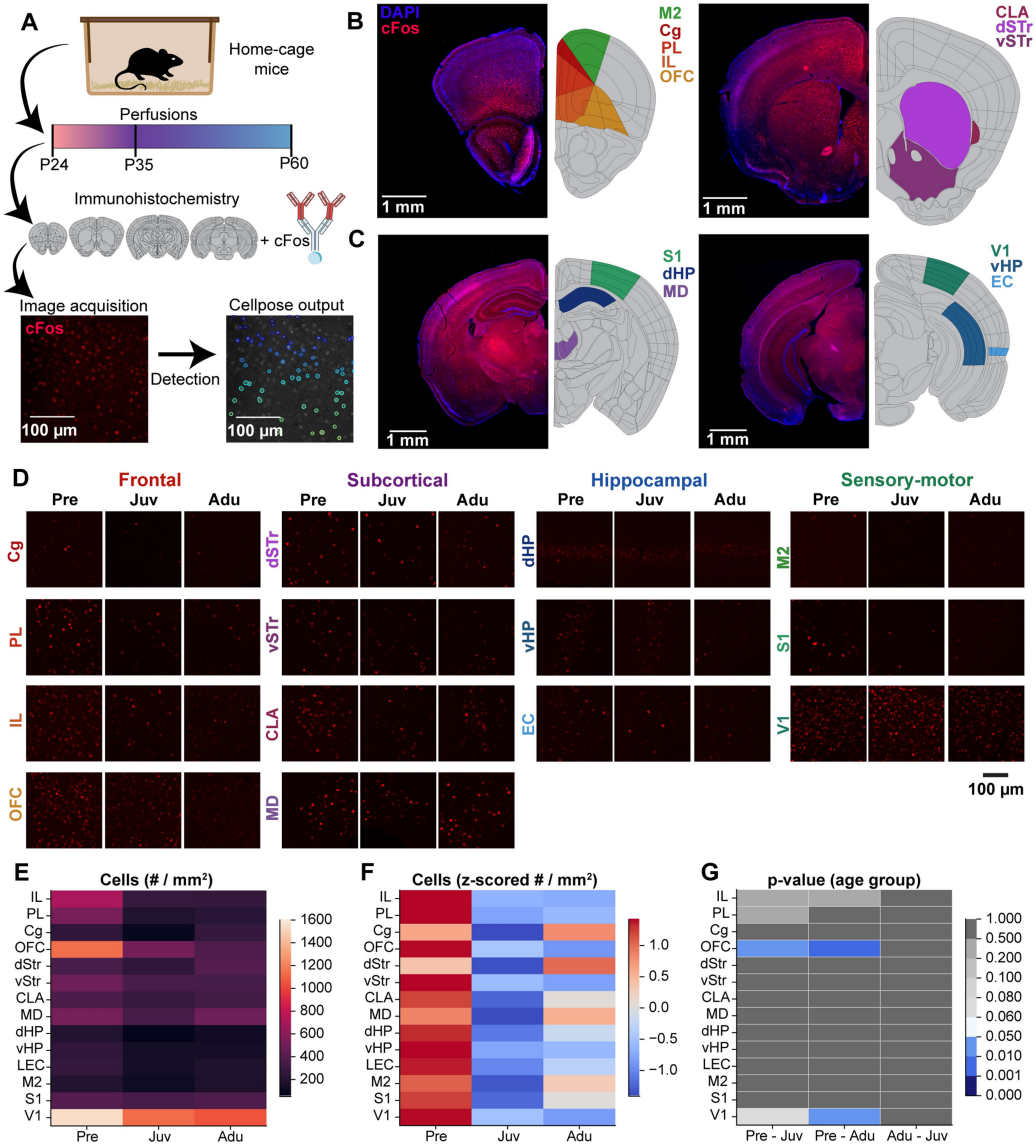
Brain-wide basal cFos expression along mouse development. **(A)** Flow chart displaying the experimental design of immunohistochemistry staining of brain slices against cFos, confocal image acquisition, and Cellpose image quantification. **(B)** Left, representative confocal images of DAPI (blue) and cFos (red) stainings (left) as well as color-coded schematic reference images from the Allen brain atlas illustrating the brain regions of interest (right, M2, Cg, PL, IL, OFC) of atlas section 37. Right, same as left for atlas section 44 including CLA, dSTR, vSTR. **(C)** Same as (B) for atlas section 74 including S1, dHP, MD (left) and for atlas section 87 including V1, vHP, EC (right). **(D)** Representative confocal images of cFos expression in frontal, subcortical, hippocampal, and sensory-motor areas from Pre, Juv, and Adu mice. **(E)** Color-coded heatmap of the number of cFos positive cells for each investigated brain area for Pre (*n* = 332 images, 4 mice), Juv (*n* = 384 images, 4 mice), and Adu (*n* = 323 images, 4 mice) mice. Data are presented as mean per brain area. **(F)** Same as **(E)** for row z-scored number of cFos positive cells. **(G)** Color-coded heatmap of statistical results of age group effects for each investigated brain area. Statistics were performed with LME models [# cells ~ age group * brain area + (1 | animal) + (1 | slice) + (1 | sex)]. See also Statistics Table S1 for detailed statistics.

To gain a first insight into the neural circuits underlying the WM and DM abilities along development, we investigated cFOS expression in multiple brain areas (Figure 4A). cFos expression was quantified in prefrontal areas [cingulate gyrus (Cg), prelimbic cortex (PL), infralimbic cortex (IL), orbitofrontal cortex (OFC)], subcortical areas [claustrum (CLA), dorsal striatum (dSTR), ventral striatum (vSTR), mediodorsal nucleus of thalamus (MD)], hippocampal areas [dorsal hippocampus (dHP), ventral hippocampus (vHP), entorhinal cortex (EC)], and sensory-motor areas [secondary motor cortex (M2), primary sensory cortex (S1), primary visual cortex (V1)] (Figures 4B–D).

In the first step, we assessed the cFos expression for mice in the home cage which did not perform the task (i.e., basal expression). The overall highest number of cFos-positive cells across age was detected for V1 and OFC. All other investigated areas showed roughly half of the numbers compared to V1. Solely, at pre-juvenile age, the IL also showed increased cFos expression (Figure 4E). Most of the investigated brain areas showed a general decrease in basal cFos expression after pre-juvenile age, which reached significance for the OFC and V1. Only the Cg and dStr showed a different trajectory and reached the highest numbers at adult age (Figures 4F,G).

### 3.5 WM and DM tasks lead to age-and brain region-specific changes in cFos expression

**Figure 5:**
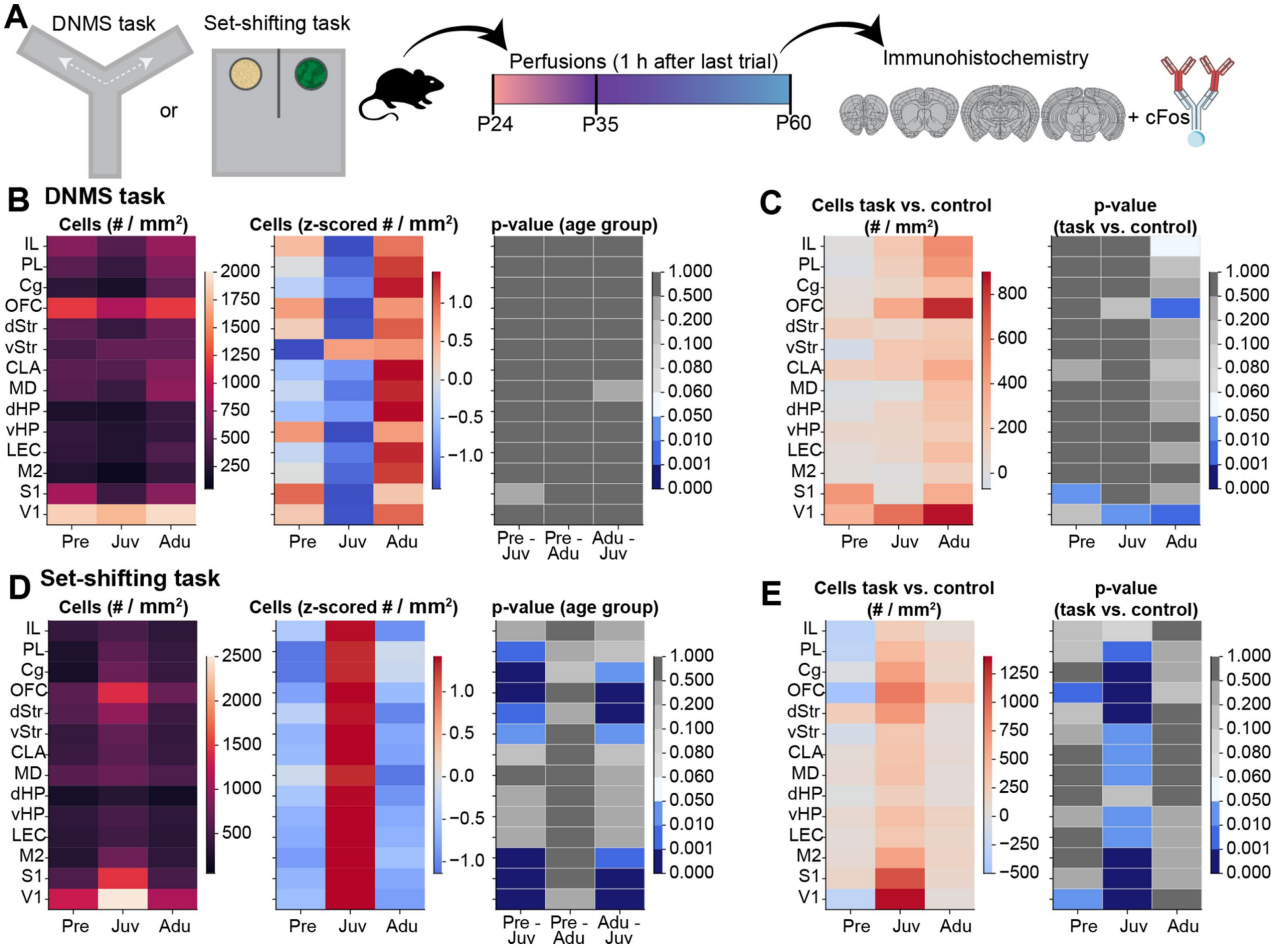
Brain-wide cFos expression after WM and DM task performance along mouse development. **(A)** Schematic displaying the experimental timeline. **(B)** Left, color-coded heatmap of the number of cFos positive cells for each investigated brain area after the last trial of the DNMS task for Pre (*n* = 347 images, 4 mice), Juv (*n* = 342 images, 4 mice), and Adu (*n* = 401 images, 4 mice) mice. Data are presented as mean per brain area. Middle, same as left for row z-scored number of cFos positive cells. Right, color-coded heatmap of statistical results of age group effects for each investigated brain area. Statistics were performed with LME models [# cells ~ age group * brain area + (1 | animal) + (1 | slice) + (1 | sex)]. **(C)** Left, color-coded heatmap of the change in cFos positive cell counts relative to control mice for each investigated brain area after the last trial of the DNMS task for Pre (*n* = 347 images, 4 mice), Juv (*n* = 342 images, 4 mice), and Adu (*n* = 401 images, 4 mice) mice. Data are presented as mean per brain area. Right, color-coded heatmap of statistical results of WM task-induced effects per age and brain area. Statistics were performed with LME models [# cells ~ treatment * age group * brain area + (1 | animal) + (1 | slice) + (1 | sex)]. **(D)** Same as (B) for cFos positive cell count after the last trial of the EDSS set-shifting phase for Pre (*n* = 433 images, 4 mice), Juv (*n* = 430 images, 4 mice), and Adu (*n* = 410 images, 4 mice) mice. **(E)** Same as **(C)** for DM task-induced effects for Pre (*n* = 433 images, 4 mice), Juv (*n* = 430 images, 4 mice), and Adu (*n* = 410 images, 4 mice) mice. See also Statistics Table S1 for detailed statistics.

Next, we investigated cFos expression changes in mice from all three age groups after performing the DNMS (WM) or set-shifting (DM) task (Figure 5A; Supplementary Figures S2A–H). In contrast to the previously observed decrease in basal cFos expression in control mice after pre-juvenile age, no differences in cFos positive cell count were detected between the investigated age groups after the DNMS task. Overall, Adu mice showed generally higher absolute cell counts than younger mice (Figure 5B). When quantifying the DNMS task-induced change in cFos-positive cells in comparison to control mice, Adu mice showed the strongest increase in prefrontal, subcortical, and hippocampal brain areas. In contrast, V1 and solely S1, showed an age-independent upregulation, whereas no changes were detected for M2. However, due to a high inter-animal variability, the task-induced upregulation only reached significance for the OFC and V1 in Adu, the V1 in Juv, and the S1 in Pre mice (Figure 5C; Supplementary Figure S3A). Hence, changes in cFos expression patterns tend to align with the behavioral findings, which identified Adu mice as the most effective in solving the task.

For the DM task, we observed a strong increase in cFos expression in Juv mice. After the last trial of the EDSS set-shifting phase, the absolute cell count in most prefrontal and sensory-motor areas, as well as in the dStr and vStr, was significantly higher in Juv compared to younger or older mice (Figure 5D). Consequently, the task-induced change in cFos expression in comparison to control mice reached significance for all investigated brain regions, except for IL and dHP, in Juv mice. In Pre mice, similar results were detected for the OFC and V1. Adu mice showed no significant upregulation and merely the OFC showed a tendency toward increased task-induced cFos expression (Figure 5E; Supplementary Figure S3B). These results suggest that the juvenile brain most strongly upregulates cFos expression during the set-shifting task, possibly contributing to faster problem-solving strategies in the Juv group.

When relating the percentage of correct trials (i.e., higher values better performance) on the last day of WM performance with the task-induced change in cFos expression, we identified a positive relationship (Supplementary Figures S4A,B). For the set-shifting task, the average latency of the EDSS phase (i.e., lower values better performance) showed a negative relationship with the change in cFos positive cell count for most of the investigated brain regions, except for the MD and dHP. Especially the cell counts in the OFC showed a significant prediction of EDSS latencies (Supplementary Figures S4C,D).

Notably, all identified differences for basal and task-dependent cFos expression showed a sex-independent trajectory (Statistics Table S2). Overall, the changes in cFos expression largely correspond to the observed behavioral performance during WM and DM and suggest that distinct neuronal mechanisms underlie age-dependent coping strategies with brain region-specific activation patterns.

## 4 Discussion

Mice represent a widely used model organism for neurodevelopmental disorders, yet the developmental trajectory of mouse cognitive abilities remains poorly understood. While mice differ from humans in terms of brain structure and complexity of cognitive abilities, they share common features of functional development that suggest a shared scheme of cognitive maturation (Carlen, 2017; Chini and Hanganu-Opatz, 2021). Determining similarities as well as differences between mouse and human cognitive development is crucial for translating the developmental mechanisms that underlie the emergence of neurodevelopmental mental disorders, such as schizophrenia. To fill this knowledge gap, the present study investigated the trajectories of WM and DM development in mice in relation to the activation of multiple brain regions monitored by cFos expression. We show that (i) optimal WM performance is present at adult age, (ii) DM abilities peak at juvenile age, (iii) cFos expression shows age-and brain area-dependent changes that relate to task performance. Moreover, we monitored the sex dependence of behavioral performance in our statistical model (see section “Materials and methods,” Statistics Table S2). In line with previous findings reporting few, if any, sex-dependent effects on adolescent-typical behaviors as well as on prefrontal activity (Pöpplau et al., 2023; Varlinskaya et al., 2013; Vetter-O’Hagen and Spear, 2012), male and female mice had similar performance in WM and DM tasks throughout development.

We observed that during SpA juvenile mice spent more time within the center than older or younger mice which may result from an increased tendency toward risk-taking behavior. However, also other reasons might account for this difference since the center zone of the Y-maze is relatively small and does not capture the complexity of risk-taking behavior. Nevertheless, augmented and seemingly irrational risk-taking behavior during juvenile age has been previously described (Spear, 2000; Steinberg, 2010) and might provide an advantage during distinct cognitive flexibility tasks (Cassotti et al., 2011). Indeed, the present results show that juvenile mice performed faster and less variable than pre-juvenile mice in the two-choice set-shifting task exclusively during the task stages requiring cognitive flexibility (IDSS, EDSS). Notably, no differences were found between pre-juvenile and adult mice, indicating that DM abilities peak at juvenile age. However, the chosen age of P31-35 might not resemble the peak maximum for increased DM abilities in juveniles. Comparing previous studies indicates that reversal learning might peak shortly before P30 and is disrupted toward P40 with the onset of puberty (Klune et al., 2021). Notably, the disruption of DM performance around P40 matches a previously reported temporal decrease in prefrontal gamma activity, indicating a reorganization of prefrontal-dependent functions at this age (Pöpplau et al., 2023). Thus, a longitudinal investigation of the whole developmental period would be necessary to determine the exact trajectories of DM performance. The present investigation contributes to this by also monitoring pre-juvenile mice. Overall, all age groups, including the youngest pre-juvenile group, were well able to learn the DM task, indicated by the overall low number of trials to reach the criterion. This might be due to the task structure, relying on textural cues and smell, a sense dominantly present and vital for survival in rodents already from birth onward (Whishaw and Kolb, 2005).

Higher flexibility in juvenile mice might be linked to the stage of structural and functional development of the PFC. Juvenile mice have been found to exhibit increased dendritic complexity and spine density as well as pronounced synaptic pruning when compared to pre-juvenile or adult mice (Johnson et al., 2016; Mallya et al., 2019; Pöpplau et al., 2023). During the same time, the PFC is influenced by the endocannabinoid system, dopamine, serotonin, and norepinephrine, all forming a complex network during development (Klune et al., 2021). Especially dopaminergic projections and receptor densities undergo major changes throughout juvenile development and reach full maturity only at the age of P35. Juvenile mice lack mature dopamine-dependent inhibitory control within the mPFC (Caballero and Tseng, 2016). Moreover, the juvenile mPFC is characterized by a peak in broadband gamma and spiking activity (Pöpplau et al., 2023). Overall, these unique circumstances in the juvenile PFC most likely contribute to more impulsive behavior, which might favor more flexible decisions over adhering to previously learned rules. Notably, a similar peak in cognitive flexibility during juvenile development has been described for humans (Crone and Dahl, 2012; Hauser et al., 2015), further emphasizing the presence of functional parallels between mice and humans.

In contrast to DM abilities, WM abilities reached mature performance only in adult mice. At this age, the task-characteristic learning curve during the time window of investigation was present. However, an increase in performance was already present between pre-and juvenile age, indicating a continuous increase in WM abilities with age. A recent study suggests that the ability to improve in WM tasks is already present in pre-juvenile rodents but requires longer and more intense training (Bevandic et al., 2024). Nonetheless, within our DNMS paradigm, all age groups performed close to or above chance level, most likely resulting from the innate alternating behavior of mice, driving them to explore novel areas over previously visited areas (Kraeuter et al., 2019). These results align with studies in humans, showing that WM modalities improve from pre-juvenile to young adult age (Conklin et al., 2007; Heled et al., 2022; Uhlhaas et al., 2009). Adults have been shown to rather stick to a learned strategy that promises long-term success at a low risk than exploring new strategies (Cassotti et al., 2011). Whereas this might represent a disadvantage during tasks involving fast DM abilities, it most likely aids them in tasks with consistent rules such as the DNMS task.

Rhythmic gamma oscillations in PFC have been shown to closely relate to WM performance and increase in power and rhythmicity with cognitive demand (Roux et al., 2012). Parvalbumin expressing interneurons are central for their generation due to a timed reciprocal interaction with excitatory neurons (Cardin et al., 2009; Chen et al., 2017). Parvalbumin expression in mPFC continues to increase throughout juvenile age and only stabilizes in early adulthood, accompanied by a general increase in inhibition strength and the emergence of mature gamma oscillations (Bitzenhofer et al., 2020; Pöpplau et al., 2023). In line with this, human studies have found more focal patterns of induced activity, higher synchrony as well as a lower signal-noise ratio during cognitive tasks in adults than in younger participants (Segalowitz et al., 2010; Uhlhaas et al., 2010; Uhlhaas et al., 2009; Durston et al., 2006). Overall, more refined patterns of activity might contribute to improved WM performance by enabling the recall of previously learned patterns more easily.

To get first insights into potential neuronal mechanisms underlying these age-dependent changes in DM and WM abilities, we quantified cFos expression changes in several brain areas. cFos is a transcription factor that controls downstream targets that drive long-term synaptic plasticity. It is a reliable biomarker of cellular activity for excitatory pathways and has been shown to get rapidly upregulated after transient behavioral stimuli (around 1 h) (Aparicio et al., 2022; Minatohara et al., 2015). Since the exact relationship between cFos expression and neuronal activity is still a matter of debate (Aparicio et al., 2022; Chung, 2015), we investigated the cFos expression to get first insights into the brain regions that are mainly involved in age-specific WM and DM abilities. The observed decline in the basal cFos expression after pre-juvenile age is in line with previous findings for frontal and hippocampal brain regions (Pompeiano and Colonnese, 2023; Jia et al., 2018). However, the steepest decrease in basal cFos expression is suggested to occur before P15 (Chen et al., 1998) and might relate to the overall sparsification of activity within the mPFC (Chini et al., 2022).

Overall, task-induced changes in cFos expression aligned with the behavioral performance. However, the DM task led to more pronounced cFos modifications than the WM task. The different task structures of the DM and WM protocol might account for this. Whereas the WM task follows a stereotypical protocol with similar repetitions on each of the 5 days, the DM task lasts only for 2 days with distinct changes in the protocol for each phase. Thus, the most pronounced changes in cFos expression may occur earlier during the testing period of the WM task and are rather minor on the last day. Moreover, changes in cFos expression with age have been suggested to foster the development of memory systems. Due to lower basal cFos expression levels, adults might require reduced upregulation in comparison to pre-or juveniles to achieve a similar gain in synaptic plasticity (Bessieres et al., 2019; Jia et al., 2018), which might explain the only moderate upregulation in Adu mice after the WM task besides their better performance. Especially the increase in cFos expression in the OFC predicted the behavioral performance during the set-shifting task. This is in line with previous reports, identifying the involvement of the OFC in the formation of attentional sets (Chase et al., 2012). Taken together, our results show that behavioral coping strategies at different stages of development relate to task-induced modifications in cFos expression, indicating age-specific underlying neuronal mechanisms.

Mouse models are instrumental in gaining knowledge about the cellular mechanisms determining the emergence of mental diseases. Most of these diseases share a developmental etiology and are caused by a combination of genetic and environmental factors that interfere with normal brain development (Feigenson et al., 2014; Horvath and Mirnics, 2009; Schubert et al., 2015). However, current treatment approaches are mainly symptomatic and only administered when the disease has already reached an advanced state (Millan et al., 2012; Millan et al., 2016; Pratt et al., 2012). Notably, cognitive symptoms and altered gamma activity are already present in prodromal cohorts, indicating altered processing before disease onset (Chini and Hanganu-Opatz, 2021; Larson et al., 2010). A mechanistic understanding of disease progression could pave the way for the identification of early biomarkers. For this, mice are particularly suited to investigate disease etiology due to the presence of vast genetic techniques and controlled housing conditions (Chini and Hanganu-Opatz, 2021). To translate findings from mice to humans, it is essential to align their developmental stages as closely as possible. Our results show that cognitive maturation in mice shows high similarities to what is known in humans, increasing the significance of translational approaches targeting developmental stages.

## 5 Concluding remarks

The investigated behavioral tasks showed distinct patterns of age-dependent developmental trajectories. Adult mice showed the most sophisticated performance during the WM task and our results suggest a constant increase in WM abilities with age. In contrast, flexibility during the DM task peaked at juvenile age, most likely supported by heightened risk-taking behavior during this period. The behavioral data relate to cFos expression changes after task performance. However, the underlying neuronal mechanisms at each investigated developmental time point that promote the observed behavioral performance need to be investigated. Future research including invasive electrophysiology during task performance might lead to a better understanding of the development of neuronal circuits enabling cognitive functioning.

## Data Availability

The original contributions presented in the study are included in the article/Supplementary material, further inquiries can be directed to the corresponding author.
