# Correction: Trajectories of working memory and decision making abilities along juvenile development in mice

**DOI:** 10.3389/fnins.2025.1684866

**Published:** 2025-09-01

**Authors:** Ann Marlene Thies, Irina Pochinok, Annette Marquardt, Maria Dorofeikova, Ileana L. Hanganu-Opatz, Jastyn A. Pöpplau

**Affiliations:** Institute of Developmental Neurophysiology, Center for Molecular Neurobiology, Hamburg Center of Neuroscience, University Medical Center Hamburg-Eppendorf, Hamburg, Germany

**Keywords:** working memory, decision making, mouse behavior, development, prefrontal cortex, cFos

In the published article, there was an error in Figure 4 as published. Two images were wrongly used in Figure 4. Figure 4D (OFC right) is same as in Figure 4D (OFC middle). Figure 4D (dSTr middle) is same as in Figure 4D (S1 left). The corrected Figure 4 and its caption appear below.

**Figure 4:**
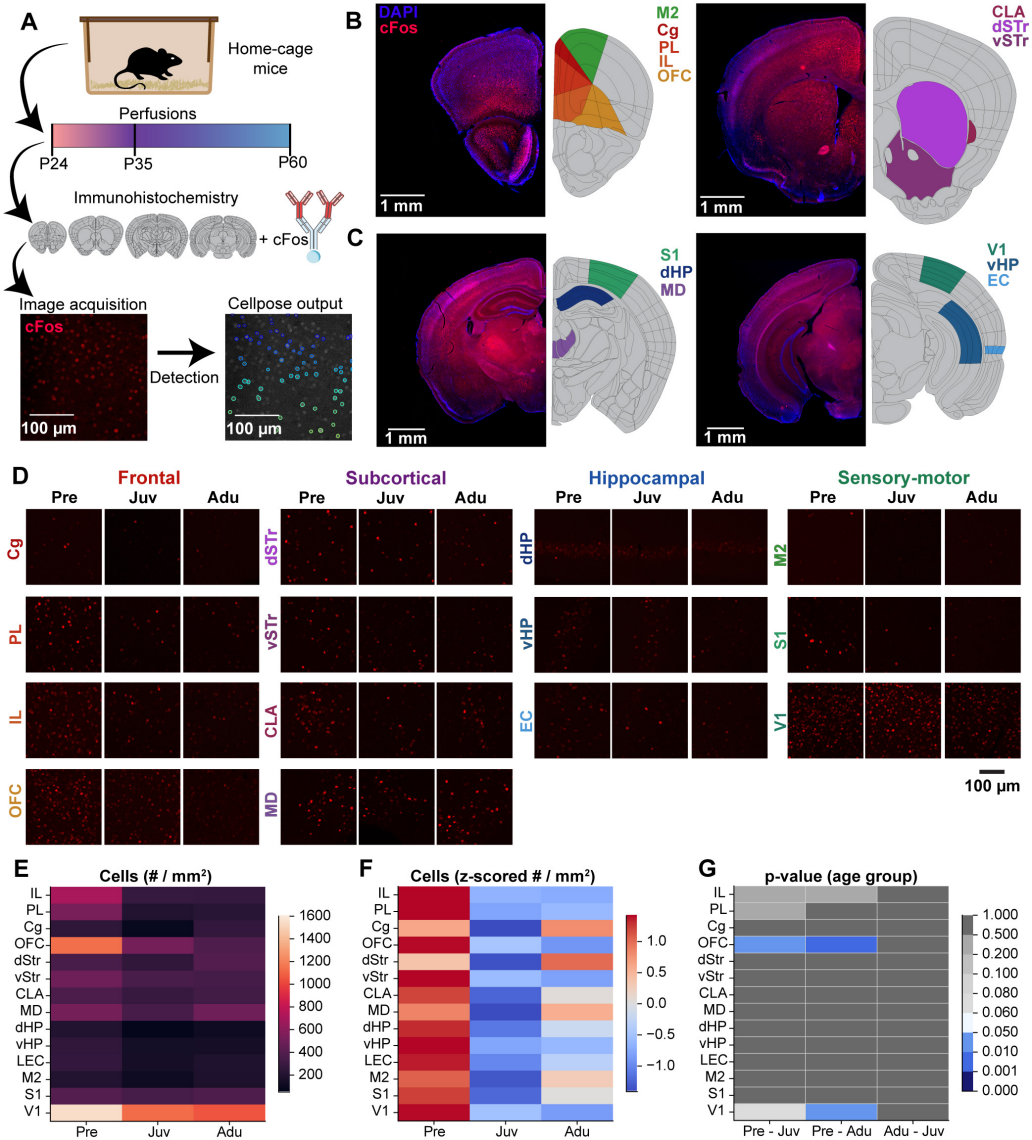
Brain-wide basal cFos expression along mouse development. **(A)** Flow chart displaying the experimental design of immunohistochemistry staining of brain slices against cFos, confocal image acquisition, and Cellpose image quantification. **(B)** Left, representative confocal images of DAPI (blue) and cFos (red) stainings (left) as well as color-coded schematic reference images from the Allen brain atlas illustrating the brain regions of interest (right, M2, Cg, PL, IL, OFC) of atlas section 37. Right, same as left for atlas section 44 including CLA, dSTR, vSTR. **(C)** Same as **(B)** for atlas section 74 including S1, dHP, MD (left) and for atlas section 87 including V1, vHP, EC (right). **(D)** Representative confocal images of cFos expression in frontal, subcortical, hippocampal, and sensory-motor areas from Pre, Juv, and Adu mice. **(E)** Color-coded heatmap of the number of cFos positive cells for each investigated brain area for Pre (*n* = 332 images, 4 mice), Juv (*n* = 384 images, 4 mice), and Adu (*n* = 323 images, 4 mice) mice. Data are presented as mean per brain area. **(F)** Same as **(E)** for row z-scored number of cFos positive cells. **(G)** Color-coded heatmap of statistical results of age group effects for each investigated brain area. Statistics were performed with LME models (# cells ~ age group ^*^ brain area + (1 | animal) + (1 | slice) + (1 | sex)). See also Statistics table S1 for detailed statistics.

The original version of this article has been updated.

