# Supplementary material for: Trajectories of working memory and decision making abilities along juvenile development in mice
Source: Front Neurosci. 2025 Feb 28;19:1524931. doi: 10.3389/fnins.2025.1524931 (PMC11906447; doi:10.3389/fnins.2025.1524931)
Supplement: Supplementary file 1 [file Data_Sheet_1.pdf]

Trajectories of working memory and decision making abilities along juvenile development in mice

Ann Marlene Thies, Irina Pochinok, Annette Marquardt, Maria Dorofeikova, Ileana L. Hanganu-Opatz & Jastyn A. Pöpplau

Inventory of Supplementary Information

Item	page
Figure S1	3
FigureS2	5
FigureS3	6
FigureS4	8
Statistics table S1	10-26
Statistics table S2	27-29

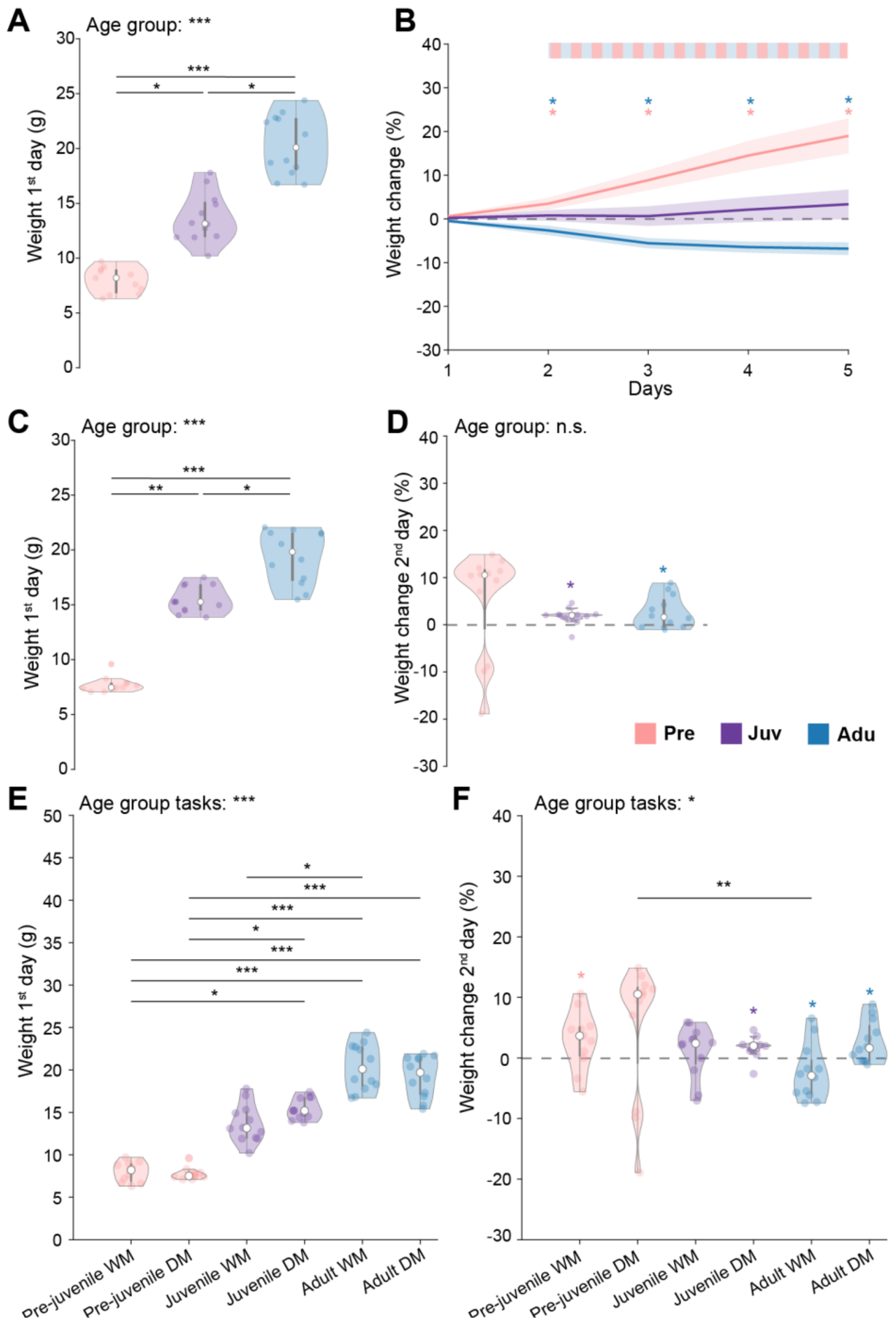

Figure S1 related to Figures 1-3. Weight and weight change quantifications along test periods. (A) Violin plots displaying the weight of Pre (n= 11), Juv (n= 12), and Adu (n= 12) mice on the first day of WM testing. **(B)** Line plots displaying the weight change from the second to the fifth day of WM testing relative to the weight on the first day. **(C)** Violin plots displaying the weight of Pre (n= 12), Juv (n = 12), and Adu (n= 12) mice on the first day of DM testing. **(D)** Same as (C) for weight change on the second day of DM testing relative to the weight on the first day. **(E)** Violin plots displaying the data of (A) and (C) for cross-task comparison of mouse weight on the first day of WM and DM testing. **(F)** Violin plots displaying the data of (B) and (D) for cross-task comparison of weight change on the second day of WM and DM testing. Black asterisks indicate significant differences between the groups (* $p < 0.05$, ** $p < 0.01$, *** p

< 0.001) and were calculated using the Kruskal-Wallis test with Bonferroni corrected post hoc. Colored single stars indicate a significant difference from zero and were calculated using the Wilcoxon signed rank test for zero median See also Statistics table S1 for detailed statistics including age group effects for single days in B.

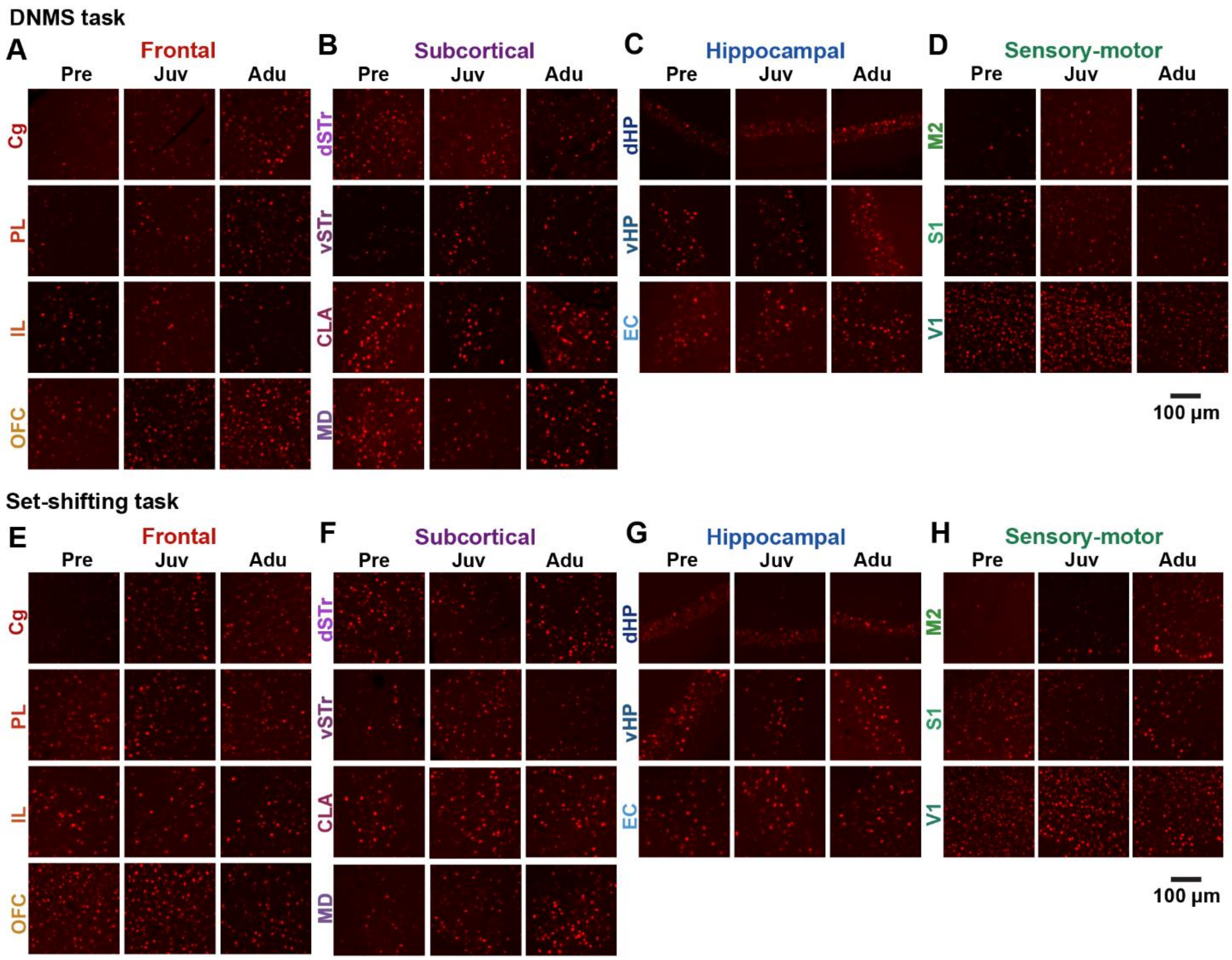

Figure S2 related to Figure 5. WM and DM task-induced changes in cFos expression along mouse development. (A) Representative confocal images of cFos expression in frontal areas from Pre, Juv, and Adu mice after the fifth day of WM testing. **(B)** Same as (A) for subcortical areas. **(C)** Same as (A) for hippocampal areas. **(D)** Same as (A) for sensory-motor areas. **(E)** Representative confocal images of cFos expression in frontal areas from Pre, Juv, and Adu mice after the second day of DM testing. **(F)** Same as (E) for subcortical areas. **(G)** Same as (E) for hippocampal areas. **(H)** Same as (E) for sensory-motor areas.

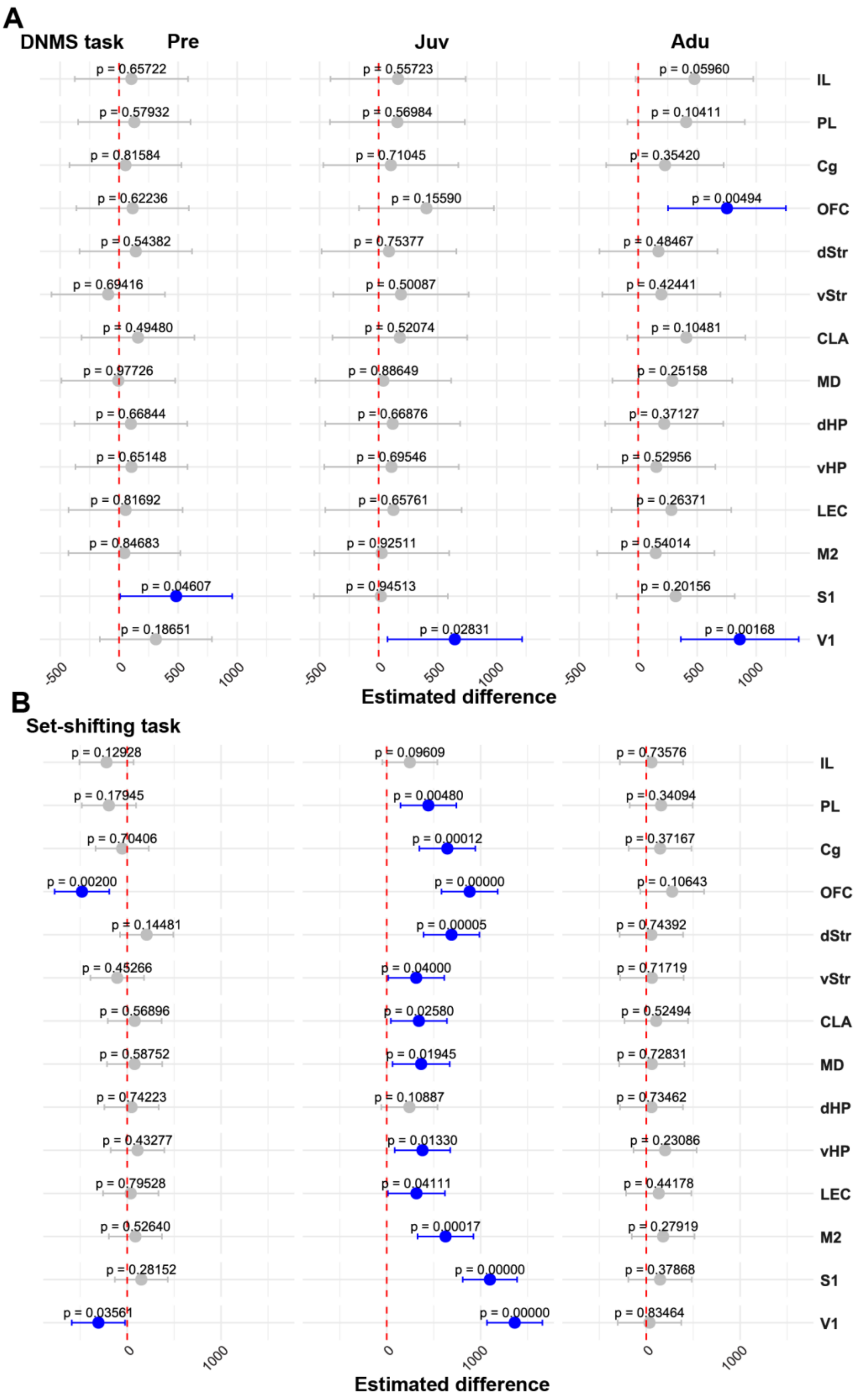

Figure S3 related to Figure 5. Summary of task-induced effects. (A) Effects and confidence interval of pairwise comparisons of the number of cFos positive cells between control mice and mice that performed the DNMS task for each investigated brain area. **(B)** same as (A) for pairwise comparisons between control

mice and mice that performed the set-shifting task. Significant results are indicated in blue. Dots indicate the estimated difference between means of the task and the control group. Horizontal lines with vertical bars indicate the 95% confidence interval. See also Statistics table S1 for detailed statistics.

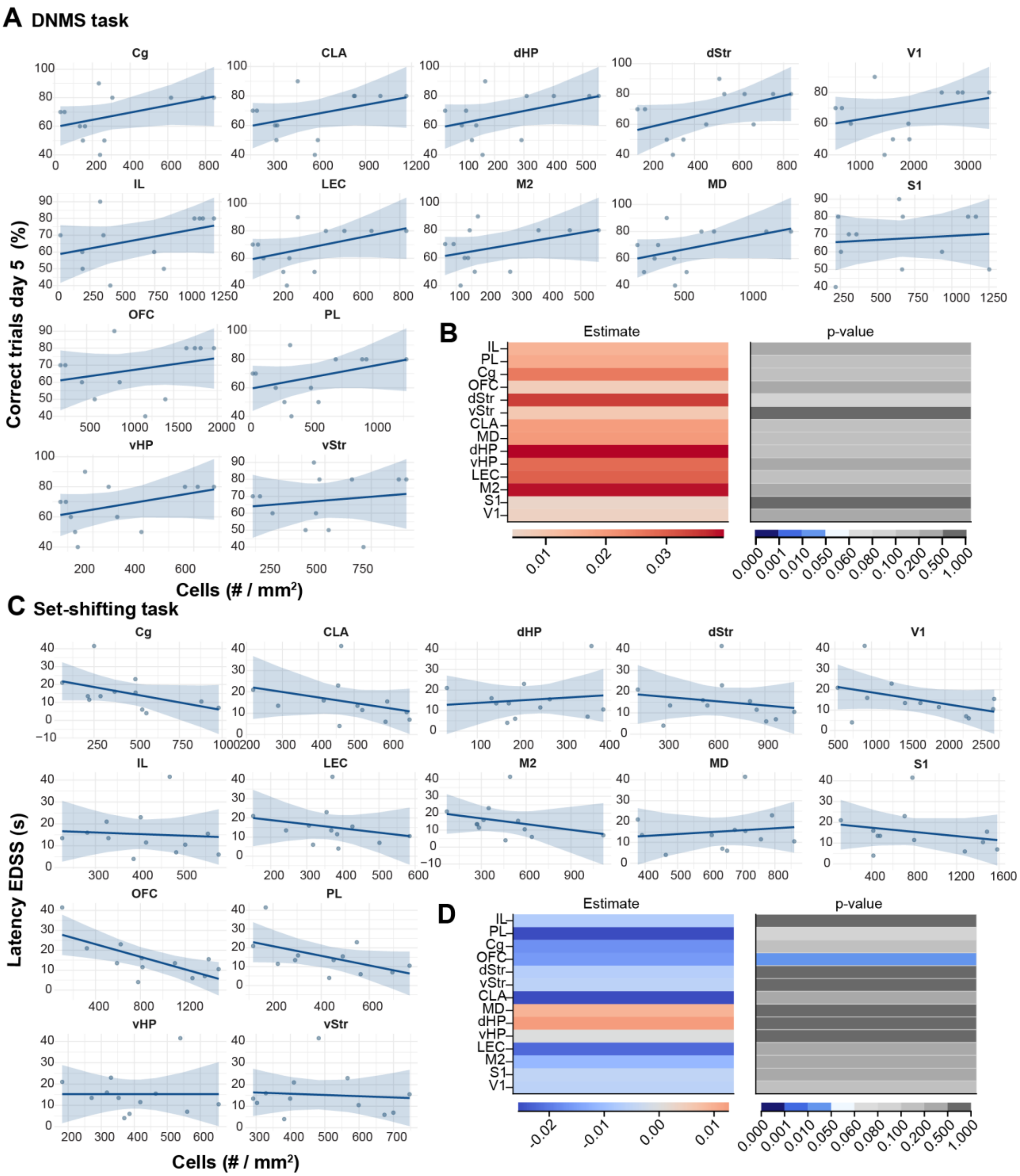

Figure S4 related to Figure 5. Relationship between DM or WM performance and cFos expression change. (A) Line plots displaying the predicted values of the percentage of correct trials in the DNMS task on day 5 as a function of task-induced cFos expression based on the linear model (performance ~ average # cells). The solid line represents the model fit. Shaded areas indicate the 95% confidence interval. Single points displaying the performance and average cFos count for each investigated mouse (n= 4 mice / age group). **(B)** Left, color-coded heatmap of the slope (estimate) of the linear model shown in (A). Right, color-coded heatmap of statistical results (significance of the slope). **(C)** Same as (A) for latencies during the

EDSS set-shifting phase (n=4 mice / age group). **(D)** Same as (B) for results shown in (C). See also Statistics table S1 for detailed statistics.

Statistics table S1. Detailed statistical results.

Figure	Test	Factor	Groups	df	Test value	p-value
**Figure 1**						
Figure 1B	Kruskal-Wallis-Test (kruskalwallis)	Age group	Entries (#)<br>Pre (11 mice)<br>Juv (12 mice)<br>Adu (12 mice)	2	Chi-Square: 21.60	2.04e-05
	Bonferroni corrected post hoc test (multcompare)		Pre-Juv<br>Pre-Adu<br>Juv-Adu			0.094<br>1.06e-05<br>0.033
Figure 1C	Kruskal-Wallis-Test (kruskalwallis)	Age group	Alternations (%)<br>Pre (11 mice)<br>Juv (12 mice)<br>Adu (12 mice)	2	Chi-Square: 3.66	0.159
Figure 1D	Kruskal-Wallis-Test (kruskalwallis)	Age group	Center (%)<br>Pre (11 mice)<br>Juv (12 mice)<br>Adu (12 mice)	2	Chi-Square: 13.06	0.001
	Bonferroni corrected post hoc test (multcompare)		Pre-Juv<br>Pre-Adu<br>Juv-Adu			0.016<br>1<br>0.002
Figure 1E	Kruskal-Wallis-Test (kruskalwallis)	Age group	Clockwise (%)<br>Pre (11 mice)<br>Juv (12 mice)<br>Adu (12 mice)	2	Chi-Square: 5.51	0.063
Figure 1F	Kruskal-Wallis-Test (kruskalwallis)	Age group	Average speed during movement (cm/s)<br>Pre (11 mice)<br>Juv (12 mice)<br>Adu (12 mice)	2	Chi-Square: 17.06	1.97e-04
	Bonferroni corrected post hoc test (multcompare)		Pre-Juv<br>Pre-Adu<br>Juv-Adu			0.051<br>1.29e-04<br>0.228
Figure 1G	Kruskal-Wallis-Test (kruskalwallis)	Age group	Total distance (m)<br>Pre (11 mice)<br>Juv (12 mice)<br>Adu (12 mice)	2	Chi-Square: 18.60	9.15e-05
	Bonferroni corrected post hoc test (multcompare)		Pre-Juv<br>Pre-Adu<br>Juv-Adu			0.019<br>5.81e-05<br>0.346
Figure 1H	Kruskal-Wallis-Test (kruskalwallis)	Age group	Movement (%)<br>Pre (11 mice)<br>Juv (12 mice)<br>Adu (12 mice)	2	Chi-Square: 19.11	7.09e-05

	Bonferroni corrected post hoc test (multcompare)		Pre-Juv<br>Pre-Adu<br>Juv-Adu			0.007<br>6.09e-05<br>0.650
**Figure 2**						
Figure 2C (left)	Kruskal-Wallis-Test (kruskalwallis)	Age group	Performance (relative to chance)<br>Day1<br>Pre (11 mice)<br>Juv (12 mice)<br>Adu (12 mice)	2	Chi-Square: 0.176	0.916
	Wilcoxon signed rank test (signrank)		Pre<br>Juv<br>Adu			0.372<br>0.003<br>0.009
	Kruskal-Wallis-Test (kruskalwallis)	Age group	Performance (relative to chance)<br>Day2<br>Pre (11 mice)<br>Juv (12 mice)<br>Adu (12 mice)	2	Chi-Square: 1.420	0.492
	Wilcoxon signed rank test (signrank)		Pre<br>Juv<br>Adu			0.093<br>0.298<br>0.076
	Kruskal-Wallis-Test (kruskalwallis)	Age group	Performance (relative to chance)<br>Day3<br>Pre (11 mice)<br>Juv (12 mice)<br>Adu (12 mice)	2	Chi-Square: 2.546	0.280
	Wilcoxon signed rank test (signrank)		Pre<br>Juv<br>Adu			0.945<br>0.083<br>0.041
	Kruskal-Wallis-Test (kruskalwallis)	Age group	Performance (relative to chance)<br>Day4<br>Pre (11 mice)<br>Juv (12 mice)<br>Adu (12 mice)	2	Chi-Square: 8.477	0.014
	Bonferroni corrected post hoc test (multcompare)		Pre-Juv<br>Pre-Adu<br>Juv-Adu			0.852<br>0.012<br>0.198
	Wilcoxon signed rank test (signrank)		Pre<br>Juv<br>Adu			0.632<br>0.107<br>0.003
	Kruskal-Wallis-Test (kruskalwallis)	Age group	Performance (relative to chance)<br>Day5<br>Pre (11 mice)<br>Juv (12 mice)<br>Adu (12 mice)	2	Chi-Square: 13.209	0.001

	Bonferroni corrected post hoc test (multcompare)		Pre-Juv<br>Pre-Adu<br>Juv-Adu			0.835<br>0.001<br>0.038
	Wilcoxon signed rank test (signrank)		Pre<br>Juv<br>Adu			0.937<br>0.023<br>4.88e-04
Figure 2C (right)	Kruskal-Wallis-Test (kruskalwallis)	Age group	Normalized slope<br>Pre (11 mice)<br>Juv (12 mice)<br>Adu (12 mice)	2	Chi-Square: 8.716	0.012
	Bonferroni corrected post hoc test (multcompare)		Pre-Juv<br>Pre-Adu<br>Juv-Adu			0.781<br>0.005<br>0.030
	Wilcoxon signed rank test (signrank)		Pre<br>Juv<br>Adu			0.147<br>0.380<br>0.006
Figure 2D (left)	Kruskal-Wallis-Test (kruskalwallis)	Age group	Time<br>Day 1<br>Pre (11 mice)<br>Juv (12 mice)<br>Adu (10 mice)	2	Chi-Square: 9.711	0.009
	Bonferroni corrected post hoc test (multcompare)		Pre-Juv<br>Pre-Adu<br>Juv-Adu			1<br>0.012<br>0.030
	Kruskal-Wallis-Test (kruskalwallis)	Age group	Time<br>Day 2<br>Pre (11 mice)<br>Juv (12 mice)<br>Adu (10 mice)	2	Chi-Square: 0.162	0.922
	Kruskal-Wallis-Test (kruskalwallis)	Age group	Time<br>Day 3<br>Pre (11 mice)<br>Juv (12 mice)<br>Adu (10 mice)	2	Chi-Square: 9.811	0.007
	Bonferroni corrected post hoc test (multcompare)		Pre-Juv<br>Pre-Adu<br>Juv-Adu			0.009<br>0.054<br>1
	Kruskal-Wallis-Test (kruskalwallis)	Age group	Time<br>Day 4<br>Pre (11 mice)<br>Juv (12 mice)<br>Adu (10 mice)	2	Chi-Square: 8.443	0.015
	Bonferroni corrected post hoc test (multcompare)		Pre-Juv<br>Pre-Adu<br>Juv-Adu			0.109<br>0.015<br>1

	Kruskal-Wallis-Test (kruskalwallis)	Age group	Time<br>Day 5<br>Pre (11 mice)<br>Juv (12 mice)<br>Adu (10 mice)	2	Chi-Square: 6.465	0.040
	Bonferroni corrected post hoc test (multcompare)		Pre-Juv<br>Pre-Adu<br>Juv-Adu			0.074<br>0.093<br>1
Figure 2D (right)	Kruskal-Wallis-Test (kruskalwallis)	Age group	Normalized slope<br>Pre (11 mice)<br>Juv (12 mice)<br>Adu (10 mice)	2	Chi-Square: 3.690	0.158
	Bonferroni corrected post hoc test (multcompare)		Pre-Juv<br>Pre-Adu<br>Juv-Adu			0.340<br>0.860<br>0.032
	Wilcoxon signed rank test (signrank)		Pre<br>Juv<br>Adu			9.77e-04<br>4.88e-04<br>0.002
**Figure 3**						
Figure 3B (top)	Kruskal-Wallis-Test (kruskalwallis)	Age group	CD trials<br>Pre (12 mice)<br>Juv (12 mice)<br>Adu (12 mice)	2	Chi-Square: 0.701	0.704
Figure 3B (middle)	Kruskal-Wallis-Test (kruskalwallis)	Age group	CD latency<br>Pre (12 mice)<br>Juv (12 mice)<br>Adu (12 mice)	2	Chi-Square: 2.771	0.250
Figure 3B (bottom)	Kruskal-Wallis-Test (kruskalwallis)	Age group	CD variance<br>Pre (12 mice)<br>Juv (12 mice)<br>Adu (12 mice)	2	Chi-Square: 4.650	0.097
Figure 3C (top)	Kruskal-Wallis-Test (kruskalwallis)	Age group	CDR trials<br>Pre (12 mice)<br>Juv (12 mice)<br>Adu (12 mice)	2	Chi-Square: 0.190	0.909
Figure 3C (middle)	Kruskal-Wallis-Test (kruskalwallis)	Age group	CDR latency<br>Pre (12 mice)<br>Juv (12 mice)<br>Adu (12 mice)	2	Chi-Square: 2.608	0.271
Figure 3C (bottom)	Kruskal-Wallis-Test (kruskalwallis)	Age group	CDR variance<br>Pre (12 mice)<br>Juv (12 mice)<br>Adu (12 mice)	2	Chi-Square: 0.415	0.812

Figure 3D (top)	Kruskal-Wallis-Test (kruskalwallis)	Age group	IDSS trials<br>Pre (12 mice)<br>Juv (12 mice)<br>Adu (12 mice)	2	Chi-Square: 0.410	0.814
Figure 3D (middle)	Kruskal-Wallis-Test (kruskalwallis)	Age group	IDSS latency<br>Pre (12 mice)<br>Juv (12 mice)<br>Adu (12 mice)	2	Chi-Square: 12.776	0.001
	Bonferroni corrected post hoc test (multcompare)		Pre-Juv<br>Pre-Adu<br>Juv-Adu			0.001<br>0.118<br>0.399
Figure 3D (bottom)	Kruskal-Wallis-Test (kruskalwallis)	Age group	IDSS variance<br>Pre (12 mice)<br>Juv (12 mice)<br>Adu (12 mice)	2	Chi-Square: 8.234	0.016
	Bonferroni corrected post hoc test (multcompare)		Pre-Juv<br>Pre-Adu<br>Juv-Adu			0.015<br>0.144<br>1
Figure 3E (top)	Kruskal-Wallis-Test (kruskalwallis)	Age group	EDSS trials<br>Pre (12 mice)<br>Juv (12 mice)<br>Adu (12 mice)	2	Chi-Square: 0.269	0.873
Figure 3E (middle)	Kruskal-Wallis-Test (kruskalwallis)	Age group	EDSS latency<br>Pre (12 mice)<br>Juv (12 mice)<br>Adu (12 mice)	2	Chi-Square: 6.913	0.031
	Bonferroni corrected post hoc test (multcompare)		Pre-Juv<br>Pre-Adu<br>Juv-Adu			0.028<br>0.297<br>1
Figure 3E (bottom)	Kruskal-Wallis-Test (kruskalwallis)	Age group	EDSS variance<br>Pre (12 mice)<br>Juv (12 mice)<br>Adu (12 mice)	2	Chi-Square: 8.789	0.012
	Bonferroni corrected post hoc test (multcompare)		Pre-Juv<br>Pre-Adu<br>Juv-Adu			0.013<br>1<br>0.092
**Figure 4**						
**Figure 4G**	Linear mixed-effects model with interaction<br><br>(# cells ~ age group * brain area + (1 | animal) + (1 | slice) + (1 | sex)	Age group<br>Brain area<br>Age group * Brain area<br><br>As random effects:<br>Animal	12 mice, 230 slices (contain multiple areas), 1039 images x brain areas.<br><br>Pre: 4 mice, 78 slices (contain multiple areas), 332 images x brain areas.<br><br>Juv: 4 mice, 76 slices (contain multiple areas), 384 images x brain areas.			

		Slice<br>Sex	Adu: 4 mice, 76 slices (contain multiple areas), 323 images x brain areas.			
	likelihood ratio test (significance of random effects Animal and Slice, effect of Sex is reported in Statistics table S2)	Animal<br>Slice		1<br>1	Chi-Square: 325.44<br>Chi-Square: 11.948	<2.2e-16<br>0.000547
	Type III Sum of Squares ANOVA	Age group<br>Brain area<br>Age group * Brain area		2<br>13<br>26	Chi-Square: 4.9362<br>Chi-Square: 691.9014<br>Chi-Square: 107.7154	0.08474<br>< 2.2e-16<br>6.474e-12
	Post-hoc Tukey-adjusted pairwise comparisons, degrees-of-freedom method: Kenward-Roger	IL:<br>Pre - Adu<br>Pre - Juv<br>Adu – Juv<br>Cg:<br>Pre - Adu<br>Pre - Juv<br>Adu - Juv<br>CLA:<br>Pre - Adu<br>Pre - Juv<br>Adu - Juv<br>dHP:<br>Pre - Adu<br>Pre - Juv<br>Adu - Juv<br>dStr:<br>Pre - Adu<br>Pre - Juv<br>Adu - Juv<br>LEC:<br>Pre - Adu<br>Pre - Juv<br>Adu - Juv<br>M2:<br>Pre - Adu<br>Pre - Juv<br>Adu - Juv<br>MD:<br>Pre - Adu<br>Pre - Juv<br>Adu - Juv	IL: 12 mice, 79 images<br>Pre: 4 mice, 21 images<br>Juv: 4 mice, 32 images<br>Adu: 4 mice, 26 images<br>Cg: 12 mice, 82 images<br>Pre: 4 mice, 28 images<br>Juv: 4 mice, 27 images<br>Adu: 4 mice, 27 images<br>CLA: 12 mice, 73 images<br>Pre: 4 mice, 23 images<br>Juv: 4 mice, 26 images<br>Adu: 4 mice, 24 images<br>dHP: 12 mice, 69 images<br>Pre: 4 mice, 20 images<br>Juv: 4 mice, 24 images<br>Adu: 4 mice, 25 images<br>dStr: 12 mice, 75 images<br>Pre: 4 mice, 26 images<br>Juv: 4 mice, 27 images<br>Adu: 4 mice, 22 images<br>LEC: 12 mice, 54 images<br>Pre: 4 mice, 18 images<br>Juv: 4 mice, 21 images<br>Adu: 4 mice, 15 images<br>M2: 12 mice, 86 images<br>Pre: 4 mice, 29 images<br>Juv: 4 mice, 29 images<br>Adu: 4 mice, 28 images<br>MD: 12 mice, 57 images<br>Pre: 4 mice, 18 images<br>Juv: 4 mice, 23 images<br>Adu: 4 mice, 16 images	 13.2<br>12.7<br>11.2<br><br>12.3<br>12.3<br>11.5<br><br>13.1<br>12.9<br>11.8<br><br>13.4<br>13.5<br>11.9<br><br>13.0<br>12.5<br>12.0<br><br>15.5<br>14.2<br>13.9<br><br>12.1<br>12.1<br>11.3<br><br>15.2<br>14.0<br>13.4	 t.ratio: 1.749<br>t.ratio: 1.828<br>t.ratio: 0.075<br><br>t.ratio: 0.006<br>t.ratio: 0.943<br>t.ratio: 1.012<br><br>t.ratio: 0.283<br>t.ratio: 0.372<br>t.ratio: 0.095<br><br>t.ratio: 0.132<br>t.ratio: 0.401<br>t.ratio: 0.295<br><br>t.ratio: -0.136<br>t.ratio: 0.542<br>t.ratio: 0.727<br><br>t.ratio: 0.475<br>t.ratio: 0.714<br>t.ratio: 0.245<br><br>t.ratio: 0.167<br>t.ratio: 0.510<br>t.ratio: 0.371<br><br>t.ratio: 0.225<br>t.ratio: 0.952<br>t.ratio: 0.772	 0.2246<br>0.2000<br>0.9969<br><br>1.0000<br>0.6246<br>0.5844<br><br>0.9569<br>0.9271<br>0.9950<br><br>0.9905<br>0.9156<br>0.9533<br><br>0.9898<br>0.8524<br>0.7524<br><br>0.8841<br>0.7591<br>0.9676<br><br>0.9847<br>0.8681<br>0.9276<br><br>0.9726<br>0.6179<br>0.7258

		OFC:<br>Pre - Adu<br>Pre - Juv<br>Adu - Juv<br>PL:<br>Pre - Adu<br>Pre - Juv<br>Adu – Juv<br>S1:<br>Pre - Adu<br>Pre - Juv<br>Adu - Juv<br>V1:<br>Pre - Adu<br>Pre - Juv<br>Adu - Juv<br>vHP:<br>Pre - Adu<br>Pre - Juv<br>Adu - Juv<br>vStr:<br>Pre - Adu<br>Pre - Juv<br>Adu - Juv	OFC: 12 mice, 72 images<br>Pre: 4 mice, 24 images<br>Juv: 4 mice, 24 images<br>Adu: 4 mice, 24 images<br>PL: 12 mice, 74 images<br>Pre: 4 mice, 21 images<br>Juv: 4 mice, 27 images<br>Adu: 4 mice, 26 images<br>S1: 12 mice, 88 images<br>Pre: 4 mice, 26 images<br>Juv: 4 mice, 40 images<br>Adu: 4 mice, 22 images<br>V1: 12 mice, 80 images<br>Pre: 4 mice, 28 images<br>Juv: 4 mice, 30 images<br>Adu: 4 mice, 22 images<br>vHP: 12 mice, 80 images<br>Pre: 4 mice, 26 images<br>Juv: 4 mice, 30 images<br>Adu: 4 mice, 24 images<br>vStr: 12 mice, 70 images<br>Pre: 4 mice, 24 images<br>Juv: 4 mice, 24 images<br>Adu: 4 mice, 22 images	 13.0<br>13.0<br>12.0<br><br>13.2<br>13.1<br>11.6<br><br>13.0<br>11.8<br>11.3<br><br>12.8<br>12.1<br>11.8<br><br>12.8<br>12.2<br>11.6<br><br>13.2<br>13.0<br>12.2	 t.ratio: 3.612<br>t.ratio: 3.070<br>t.ratio: -0.586<br><br>t.ratio: 0.984<br>t.ratio: 1.301<br>t.ratio: 0.346<br><br>t.ratio: 0.171<br>t.ratio: 0.203<br>t.ratio: 0.031<br><br>t.ratio: 2.749<br>t.ratio: 2.457<br>t.ratio: -0.352<br><br>t.ratio: 0.620<br>t.ratio: 0.599<br>t.ratio: -0.028<br><br>t.ratio: 0.805<br>t.ratio: 0.686<br>t.ratio: -0.130	 0.0083<br>0.0228<br>0.8300<br><br>0.5992<br>0.4191<br>0.9364<br><br>0.9841<br>0.9776<br>0.9995<br><br>0.0414<br>0.0720<br>0.9345<br><br>0.8119<br>0.8232<br>0.9996<br><br>0.7066<br>0.7754<br>0.9907
**Figure 5**						
**Figure 5B (right)**	Linear mixed-effects model with interaction<br><br>(# cells ~ age group * brain area + (1 | animal) + (1 | slice) + (1 | sex)	Age group<br>Brain area<br>Age group * Brain area<br><br>As random effects:<br>Animal<br>Slice<br>Sex	12 mice, 237 slices (contain multiple areas), 1090 images x brain areas.<br><br>Pre: 4 mice, 78 slices (contain multiple areas), 347 images x brain areas.<br><br>Juv: 4 mice, 78 slices (contain multiple areas), 342 images x brain areas.<br><br>Adu: 4 mice, 81 slices (contain multiple areas), 401 images x brain areas.			
	likelihood ratio test (significance of random effects Animal and Slice, effect of Sex is reported in Statistics table S2)	Animal<br>Slice		1<br>1	Chi-Square: 400.97<br>Chi-Square: 23.195	< 2.2e-16<br>1.464e-06
	Type III Sum of Squares ANOVA	Age group<br>Brain area		2<br>13<br>26	Chi-Square: 1.7685<br>Chi-Square: 718.6367<br>Chi-Square: 55.2536	0.4130326<br>< 2.2e-16<br>0.0007038

		Age group * Brain area				
	Post-hoc Tukey-adjusted pairwise comparisons, degrees-of-freedom method: Kenward-Roger	IL:	IL: 12 mice, 72 images			
		Pre - Adu	Pre: 4 mice, 22 images	9.79	t.ratio: -0.189	0.9804
		Pre - Juv	Juv: 4 mice, 20 images	11.42	t.ratio: 0.904	0.6488
		Adu – Juv	Adu: 4 mice, 30 images	11.08	t.ratio: 1.072	0.5495
		Cg:	Cg: 12 mice, 79 images			
		Pre - Adu	Pre: 4 mice, 26 images	9.64	t.ratio: -0.662	0.7902
		Pre - Juv	Juv: 4 mice, 24 images	10.96	t.ratio: 0.399	0.9166
		Adu - Juv	Adu: 4 mice, 29 images	10.85	t.ratio: 0.970	0.6098
		CLA:	CLA: 12 mice, 78 images			
		Pre - Adu	Pre: 4 mice, 23 images	9.80	t.ratio: -0.739	0.7469
		Pre - Juv	Juv: 4 mice, 27 images	10.98	t.ratio: 0.152	0.9874
		Adu - Juv	Adu: 4 mice, 28 images	10.75	t.ratio: 0.793	0.7147
		dHP:	dHP: 12 mice, 83 images			
		Pre - Adu	Pre: 4 mice, 30 images	9.65	t.ratio: -0.376	0.9256
		Pre - Juv	Juv: 4 mice, 28 images	10.64	t.ratio: 0.171	0.9841
		Adu - Juv	Adu: 4 mice, 25 images	10.84	t.ratio: 0.495	0.8753
		dStr:	dStr: 12 mice, 78 images			
		Pre - Adu	Pre: 4 mice, 24 images	9.72	t.ratio: -0.221	0.9735
		Pre - Juv	Juv: 4 mice, 25 images	11.01	t.ratio: 0.493	0.8759
		Adu - Juv	Adu: 4 mice, 29 images	10.80	t.ratio: 0.686	0.7763
		LEC:	LEC: 12 mice, 63 images			
		Pre - Adu	Pre: 4 mice, 20 images	10.06	t.ratio: -0.495	0.8755
		Pre - Juv	Juv: 4 mice, 17 images	11.83	t.ratio: 0.213	0.9754
		Adu - Juv	Adu: 4 mice, 26 images	11.51	t.ratio: 0.640	0.8016
		M2:	M2: 12 mice, 83 images			
		Pre - Adu	Pre: 4 mice, 26 images	9.55	t.ratio: -0.305	0.9502
		Pre - Juv	Juv: 4 mice, 25 images	10.91	t.ratio: 0.362	0.9306
		Adu - Juv	Adu: 4 mice, 32 images	10.71	t.ratio: 0.627	0.8088
		MD:	MD: 12 mice, 64 images			
		Pre - Adu	Pre: 4 mice, 22 images	10.11	t.ratio:-0.938	0.6300
		Pre - Juv	Juv: 4 mice, 20 images	11.42	t.ratio: 0.432	0.9033
		Adu - Juv	Adu: 4 mice, 22 images	11.42	t.ratio: 1.242	0.4535
		OFC:	OFC: 12 mice, 80 images			
		Pre - Adu	Pre: 4 mice, 26 images	9.67	t.ratio: 0.159	0.9861
		Pre - Juv	Juv: 4 mice, 26 images	10.87	t.ratio: 0.892	0.6564
		Adu - Juv	Adu: 4 mice, 28 images	10.80	t.ratio: 0.755	0.7371
		PL:	PL: 12 mice, 87 images			
		Pre - Adu	Pre: 4 mice, 30 images	9.40	t.ratio:-0.387	0.9215
		Pre - Juv	Juv: 4 mice, 24 images	10.82	t.ratio: 0.694	0.7720
		Adu – Juv	Adu: 4 mice, 33 images	10.73	t.ratio: 1.027	0.5765
		S1:	S1: 12 mice, 87 images			
		Pre - Adu	Pre: 4 mice, 28 images	9.53	t.ratio: 0.762	0.7340
		Pre - Juv	Juv: 4 mice, 29 images	10.68	t.ratio: 1.634	0.2742

		Adu - Juv	Adu: 4 mice, 30 images	10.61	t.ratio: 0.979	0.6052
		V1:	V1: 12 mice, 86 images			
		Pre - Adu	Pre: 4 mice, 26 images	9.55	t.ratio:-0.079	0.9965
		Pre - Juv	Juv: 4 mice, 28 images	10.79	t.ratio: 0.406	0.9140
		Adu - Juv	Adu: 4 mice, 32 images	10.58	t.ratio: 0.476	0.8841
		vHP:	vHP: 12 mice, 81 images			
		Pre - Adu	Pre: 4 mice, 26 images	9.67	t.ratio: 0.271	0.9604
		Pre - Juv	Juv: 4 mice, 27 images	10.83	t.ratio: 0.344	0.9373
		Adu - Juv	Adu: 4 mice, 28 images	10.75	t.ratio: 0.110	0.9934
		vStr:	vStr: 12 mice, 69 images			
		Pre - Adu	Pre: 4 mice, 18 images	10.10	t.ratio:-0.531	0.8580
		Pre - Juv	Juv: 4 mice, 22 images	11.59	t.ratio:-0.506	0.8700
		Adu - Juv	Adu: 4 mice, 29 images	10.97	t.ratio:-0.048	0.9987
**Figure 5C (right)**	Linear mixed-effects model with interaction<br><br>(# cells ~ treatment * age group * brain area + (1 | animal) + (1 | slice) + (1 | sex)	Age group<br>Brain area<br>Treatment<br>Treatment * Age group * Brain area<br><br>As random effects:<br>Animal<br>Slice<br>Sex	All mice from Figure 5B (right) as Treatment group<br>All mice from Figure 4G as Control group			
	likelihood ratio test (significance of random effects Animal and Slice, effect of Sex is reported in Statistics table S2)	Animal<br>Slice		1<br>1	Chi-Square: 841.51<br>Chi-Square: 80.016	< 2.2e-16<br>< 2.2e-16
	Type III Sum of Squares ANOVA	Age group<br>Brain area<br>Treatment<br>Treatment * Age group * Brain area		2<br>13<br>1<br>26	Chi-Square: 2.7427<br>Chi-Square: 618.5398<br>Chi-Square: 0.2022<br>Chi-Square: 63.6350	0.2537609<br>< 2.2e-16<br>0.6529213<br>5.342e-05
	Post-hoc Tukey-adjusted pairwise comparisons,	 Pre:IL<br>Adu:IL<br>Juv:IL<br>Pre:Cg	All mice from Figure 5D (right) as Treatment group<br>All mice from Figure 4G as Control group	 22.50<br>22.00<br>21.42<br>21.19	t.ratio: :<br>-0.4497<br>-1.9863<br>-0.5963<br>-0.2358	 0.6572<br>0.0596<br>0.5572<br>0.8158

	degrees-of-freedom method: Kenward-Roger	Adu:Cg		21.98	-0.9465	0.3542
		Juv:Cg		21.22	-0.3763	0.7105
		Pre:CLA		22.04	-0.6942	0.4948
		Adu:CLA		22.40	-1.6905	0.1048
		Juv:CLA		21.01	-0.6532	0.5207
		Pre:dHP		21.83	-0.4342	0.6684
		Adu:dHP		22.59	-0.9123	0.3713
		Juv:dHP		21.14	-0.4339	0.6688
		Pre:dStr		21.59	-0.6168	0.5438
		Adu:dStr		22.57	-0.7105	0.4847
		Juv:dStr		21.16	-0.3178	0.7538
		Pre:LEC		23.35	-0.2341	0.8169
		Adu:LEC		24.43	-1.1440	0.2637
		Juv:LEC		23.14	-0.4490	0.6576
		Pre:M2		21.11	-0.1955	0.8468
		Adu:M2		21.66	-0.6225	0.5401
		Juv:M2		20.95	-0.0951	0.9251
		Pre:MD		23.03	0.0288	0.9773
		Adu:MD		24.59	-1.1742	0.2516
		Juv:MD		22.22	-0.1444	0.8865
		Pre:OFC		21.62	-0.4997	0.6224
		Adu:OFC		22.41	-3.1176	0.0049
		Juv:OFC		21.32	-1.4710	0.1559
		Pre:PL		21.71	-0.5628	0.5793
		Adu:PL		21.79	-1.6961	0.1041
		Juv:PL		21.22	-0.5773	0.5698
		Pre:S1		21.22	-2.1188	0.0461
		Adu:S1		22.49	-1.3156	0.2016
		Juv:S1		20.00	-0.0697	0.9451
		Pre:V1		21.19	-1.3652	0.1865
		Adu:V1		22.36	-3.5693	0.0017
		Juv:V1		20.59	-2.3588	0.0283
		Pre:vHP		21.37	-0.4581	0.6515
		Adu:vHP		22.39	-0.6386	0.5296
		Juv:vHP		20.68	-0.3970	0.6955
		Pre:vStr		22.77	0.3982	0.6942
		Adu:vStr		22.57	-0.8135	0.4244
		Juv:vStr		21.81	-0.6845	0.5009
**Figure 5D (right)**	Linear mixed-effects model with interaction<br><br>(# cells ~ age group * brain area + (1 | animal) + (1 | slice) + (1 | sex)	Age group<br>Brain area<br>Age group * Brain area<br><br>As random effects:	12 mice, 239 slices (contain multiple areas), 1273 images x brain areas.<br><br>Pre: 4 mice, 77 slices (contain multiple areas), 433 images x brain areas.<br><br>Juv: 4 mice, 83 slices (contain multiple areas), 430 images x brain areas.			

		Animal<br>Slice<br>Sex	Adu: 4 mice, 79 slices (contain multiple areas), 410 images x brain areas.			
	likelihood ratio test (significance of random effects Animal and Slice, effect of Sex is reported in Statistics table S2)	Animal<br>Slice		1<br>1	Chi-Square: 85.267<br>Chi-Square: 13.927	< 2.2e-16<br>0.0001901
	Type III Sum of Squares ANOVA	Age group<br>Brain area<br>Age group * Brain area		2<br>13<br>26	Chi-Square: 3.5862<br>Chi-Square: 524.7108<br>Chi-Square: 635.5152	0.1664<br>< 2.2e-16<br>< 2.2e-16
	Post-hoc Tukey-adjusted pairwise comparisons, degrees-of-freedom method: Kenward-Roger	IL:<br>Pre - Adu<br>Pre - Juv<br>Adu – Juv<br>Cg:<br>Pre - Adu<br>Pre - Juv<br>Adu - Juv<br>CLA:<br>Pre - Adu<br>Pre - Juv<br>Adu - Juv<br>dHP:<br>Pre - Adu<br>Pre - Juv<br>Adu - Juv<br>dStr:<br>Pre - Adu<br>Pre - Juv<br>Adu - Juv<br>LEC:<br>Pre - Adu<br>Pre - Juv<br>Adu - Juv<br>M2:<br>Pre - Adu<br>Pre - Juv<br>Adu - Juv<br>MD:<br>Pre - Adu<br>Pre - Juv	IL: 12 mice, 97 images<br>Pre: 4 mice, 33 images<br>Juv: 4 mice, 36 images<br>Adu: 4 mice, 28 images<br>Cg: 12 mice, 95 images<br>Pre: 4 mice, 32 images<br>Juv: 4 mice, 29 images<br>Adu: 4 mice, 34 images<br>CLA: 12 mice, 87 images<br>Pre: 4 mice, 32 images<br>Juv: 4 mice, 29 images<br>Adu: 4 mice, 26 images<br>dHP: 12 mice, 92 images<br>Pre: 4 mice, 30 images<br>Juv: 4 mice, 30 images<br>Adu: 4 mice, 32 images<br>dStr: 12 mice, 93 images<br>Pre: 4 mice, 34 images<br>Juv: 4 mice, 30 images<br>Adu: 4 mice, 29 images<br>LEC: 12 mice, 74 images<br>Pre: 4 mice, 24 images<br>Juv: 4 mice, 26 images<br>Adu: 4 mice, 24 images<br>M2: 12 mice, 95 images<br>Pre: 4 mice, 34 images<br>Juv: 4 mice, 29 images<br>Adu: 4 mice, 32 images<br>MD: 12 mice, 71 images<br>Pre: 4 mice, 26 images<br>Juv: 4 mice, 23 images	 22.2<br>18.0<br>21.6<br><br>20.7<br>19.7<br>21.6<br><br>23.1<br>19.7<br>24.0<br><br>21.7<br>20.0<br>21.7<br><br>21.6<br>19.1<br>22.6<br><br>26.9<br>23.3<br>26.1<br><br>20.7<br>19.3<br>22.1<br><br>27.2<br>23.8	 t.ratio: 0.520<br>t.ratio:-1.279<br>t.ratio:-1.651<br><br>t.ratio:-1.846<br>t.ratio:-5.382<br>t.ratio:-3.027<br><br>t.ratio: 0.242<br>t.ratio:-1.988<br>t.ratio:-1.998<br><br>t.ratio: 0.147<br>t.ratio:-1.196<br>t.ratio:-1.227<br><br>t.ratio: 1.228<br>t.ratio:-3.942<br>t.ratio:-4.705<br><br>t.ratio:-0.036<br>t.ratio:-1.402<br>t.ratio:-1.232<br><br>t.ratio:-0.534<br>t.ratio:-4.590<br>t.ratio:-3.576<br><br>t.ratio: 0.550<br>t.ratio:-1.072	

		Adu - Juv	Adu: 4 mice, 22 images	28.7	t.ratio:-1.499	
		OFC:	OFC: 12 mice, 78 images			
		Pre - Adu	Pre: 4 mice, 23 images	27.1	t.ratio:-0.763	
		Pre - Juv	Juv: 4 mice, 30 images	22.6	t.ratio:-7.833	
		Adu - Juv	Adu: 4 mice, 25 images	24.4	t.ratio:-6.349	
		PL:	PL: 12 mice, 93 images			
		Pre - Adu	Pre: 4 mice, 31 images	21.7	t.ratio:-1.552	
		Pre - Juv	Juv: 4 mice, 31 images	19.5	t.ratio:-4.114	
		Adu – Juv	Adu: 4 mice, 31 images	21.7	t.ratio:-2.148	
		S1:	S1: 12 mice, 115 images			
		Pre - Adu	Pre: 4 mice, 40 images	19.4	t.ratio: 0.378	
		Pre - Juv	Juv: 4 mice, 42 images	15.9	t.ratio:-9.923	
		Adu - Juv	Adu: 4 mice, 33 images	19.1	t.ratio:-9.082	
		V1:	V1: 12 mice, 95 images			
		Pre - Adu	Pre: 4 mice, 31 images	21.4	t.ratio: 1.558	
		Pre - Juv	Juv: 4 mice, 32 images	19.2	t.ratio:12.658	
		Adu - Juv	Adu: 4 mice, 32 images	21.2	t.ratio:12.954	
		vHP:	vHP: 12 mice, 94 images			
		Pre - Adu	Pre: 4 mice, 30 images	21.7	t.ratio: 0.145	
		Pre - Juv	Juv: 4 mice, 32 images	19.5	t.ratio:-1.643	
		Adu - Juv	Adu: 4 mice, 32 images	21.2	t.ratio:-1.630	
		vStr:	vStr: 12 mice, 94 images			
		Pre - Adu	Pre: 4 mice, 33 images	21.5	t.ratio:-0.090	
		Pre - Juv	Juv: 4 mice, 31 images	19.1	t.ratio:-2.985	
		Adu - Juv	Adu: 4 mice, 30 images	22.1	t.ratio:-2.564	
**Figure 5E (right)**	Linear mixed-effects model with interaction<br><br>(# cells ~ treatment * age group * brain area + (1 | animal) + (1 | slice) + (1 | sex)	Age group<br>Brain area<br>Treatment<br>Treatment * Age group * Brain area<br><br>As random effects:<br>Animal<br>Slice<br>Sex	All mice from Figure 5D (right) as Treatment group<br>All mice from Figure 4G as Control group			
	likelihood ratio test (significance of random effects Animal and Slice, effect of Sex is reported in Statistics table S2)	Animal<br>Slice		1<br>1	Chi-Square: 478.38<br>Chi-Square: 9.6423	< 2.2e-16<br>0.001901

	Type III Sum of Squares ANOVA	Age group		2	Chi-Square: 7.5101	0.02340
		Brain area		13	Chi-Square: 785.5718	< 2.2e-16
		Treatment		1	Chi-Square: 2.4359	0.11859
		Treatment * Age group * Brain area		26	Chi-Square: 331.7374	< 2.2e-16
	Post-hoc Tukey-adjusted pairwise comparisons, degrees-of-freedom method: Kenward-Roger		All mice from Figure 5D (right) as Treatment group<br>All mice from Figure 4G as Control group		t.ratio	
		Pre:IL		29.48	1.5607	0.1293
		Adu:IL		28.37	-0.3408	0.7358
		Juv:IL		26.99	-1.7243	0.0961
		Pre:Cg		27.27	0.3839	0.7041
		Adu:Cg		26.94	-0.9085	0.3717
		Juv:Cg		29.43	-4.4270	0.0001
		Pre:CLA		28.76	-0.5762	0.5690
		Adu:CLA		29.51	-0.6434	0.5249
		Juv:CLA		29.71	-2.3467	0.0258
		Pre:dHP		30.47	-0.3319	0.7422
		Adu:dHP		27.69	-0.3424	0.7346
		Juv:dHP		30.14	-1.6522	0.1089
		Pre:dStr		27.48	-1.5008	0.1448
		Adu:dStr		29.44	-0.3298	0.7439
		Juv:dStr		29.22	-4.7457	0.0001
		Pre:LEC		33.26	-0.2615	0.7953
		Adu:LEC		35.08	-0.7780	0.4418
		Juv:LEC		32.39	-2.1272	0.0411
		Pre:M2		26.70	-0.6419	0.5264
		Adu:M2		27.02	-1.1043	0.2792
		Juv:M2		28.95	-4.3126	0.0002
		Pre:MD		32.62	-0.5479	0.5875
		Adu:MD		35.05	-0.3502	0.7283
		Juv:MD		32.58	-2.4584	0.0194
		Pre:OFC		30.85	3.3762	0.0020
		Adu:OFC		29.81	-1.6648	0.1064
		Juv:OFC		30.15	-6.0256	< 0.0001
		Pre:PL		29.91	1.3747	0.1794
		Adu:PL		27.68	-0.9690	0.3409
		Juv:PL		28.99	-3.0541	0.0048
		Pre:S1		26.71	-1.0991	0.2815
		Adu:S1		28.63	-0.8942	0.3787
		Juv:S1		25.10	-7.8147	< 0.0001
		Pre:V1		27.46	2.2101	0.0356
		Adu:V1		28.72	-0.2107	0.8346
		Juv:V1		28.04	-9.4537	< 0.0001
		Pre:vHP		28.19	-0.7959	0.4328

		Adu:vHP<br>Juv:vHP<br>Pre:vStr<br>Adu:vStr<br>Juv:vStr		28.06<br>28.04<br>28.24<br>29.22<br>29.95	-1.2247<br>-2.6428<br>0.7615<br>-0.3657<br>-2.1472	0.2309<br>0.0133<br>0.4527<br>0.7172<br>0.0400
**Figure S1**						
Figure S1A	Kruskal-Wallis-Test (kruskalwallis)	Condition	Weight 1st day<br>Pre (11 mice)<br>Juv (12 mice)<br>Adu (12 mice)	2	Chi-Square: 29.240	4.47e-07
	Bonferroni corrected post hoc test (multcompare)		Pre-Juv<br>Pre-Adu<br>Juv-Adu			0.016<br>1.92e-07<br>0.021
Figure S1B	Kruskal-Wallis-Test (kruskalwallis)	Age group	Weight change day 2<br>Pre (11 mice)<br>Juv (12 mice)<br>Adu (12 mice)	2	Chi-Square: 6.315	0.043
	Bonferroni corrected post hoc test (multcompare)		Pre-Juv<br>Pre-Adu<br>Juv-Adu			1<br>0.041<br>0.301
	Wilcoxon signed rank test (signrank)		Pre<br>Juv<br>Adu			0.105<br>0.365<br>0.092
	Kruskal-Wallis-Test (kruskalwallis)	Age group	Weight change day 3<br>Pre (11 mice)<br>Juv (12 mice)<br>Adu (12 mice)	2	Chi-Square: 16.949	2.09e-04
	Bonferroni corrected post hoc test (multcompare)		Pre-Juv<br>Pre-Adu<br>Juv-Adu			0.169<br>1.20e-04<br>0.073
	Wilcoxon signed rank test (signrank)		Pre<br>Juv<br>Adu			0.009<br>0.791<br>4.88e-04
	Kruskal-Wallis-Test (kruskalwallis)	Age group	Weight change day 4<br>Pre (11 mice)<br>Juv (12 mice)<br>Adu (12 mice)	2	Chi-Square: 16,255	2.95e-04
	Bonferroni corrected post hoc test (multcompare)		Pre-Juv<br>Pre-Adu<br>Juv-Adu			0.123<br>1.67e-04<br>0.126
	Wilcoxon signed rank test (signrank)		Pre<br>Juv<br>Adu			0.005<br>0.380<br>4.88e-04

	Kruskal-Wallis-Test (kruskalwallis)	Age group	Weight change day 5<br>Pre (11 mice)<br>Juv (12 mice)<br>Adu (12 mice)	2	Chi-Square: 18.815	8.21e-05
	Bonferroni corrected post hoc test (multcompare)		Pre-Juv<br>Pre-Adu<br>Juv-Adu			0.076<br>4.32e-05<br>0.094
	Wilcoxon signed rank test (signrank)		Pre<br>Juv<br>Adu			0.003<br>0.301<br>4.88e-04
Figure S1C	Kruskal-Wallis-Test (kruskalwallis)	Age group	Weight 1st day<br>Pre (11 mice)<br>Juv (12 mice)<br>Adu (12 mice)	2	Chi-Square: 29.164	4.65e-07
	Bonferroni corrected post hoc test (multcompare)		Pre-Juv<br>Pre-Adu<br>Juv-Adu			0.008<br>2.11e-07<br>0.048
Figure S1D	Kruskal-Wallis-Test (kruskalwallis)	Age group	Weight change 2nd day<br>Pre (11 mice)<br>Juv (12 mice)<br>Adu (12 mice)	2	Chi-Square: 5.520	0.063
	Wilcoxon signed rank test (signrank)		Pre<br>Juv<br>Adu			0.109<br>0.021<br>0.042
Figure S1E	Kruskal-Wallis-Test (kruskalwallis)	Age group tasks	Weight 1st day<br>Pre WM (11 mice)<br>Pre DM (12 mice)<br>Juv WM (12 mice)<br>Juv DM (12 mice)<br>Adu WM (12 mice)<br>Adu DM (12 mice)	5	Chi-Square:	1.19e-11

	Bonferroni corrected		Pre WM-Pre DM			1
	post hoc test		Pre WM-Juv WM			0.200
	(multcompare)		Pre WM-Juv DM			0.022
			Pre WM-Adu WM			4.16e-07
			Pre WM-Adu DM			3.93e-06
			Pre DM- Juv WM			0.143
			Pre DM- Juv DM			0.013
			Pre DM- Adu WM			1.40e-07
			Pre DM- Adu DM			1.50e-06
			Juv WM-Juv DM			1
			Juv WM-Adu WM			0.024
			Juv WM-Adu DM			0.093
			Juv DM-Adu WM			0.227
			Juv DM-Adu DM			0.660
			Adu WM-Adu DM			1
Figure S1D	Kruskal-Wallis-Test	Age group	Weight 1st day	5	Chi-Square:	0.016
	(kruskalwallis)	tasks	Pre WM (11 mice)			
			Pre DM (12 mice)			
			Juv WM (12 mice)			
			Juv DM (12 mice)			
			Adu WM (12 mice)			
			Adu DM (12 mice)			
	Bonferroni corrected		Pre WM-Pre DM			1
	post hoc test		Pre WM-Juv WM			1
	(multcompare)		Pre WM-Juv DM			1
			Pre WM-Adu WM			0.220
			Pre WM-Adu DM			1
			Pre DM- Juv WM			0.976
			Pre DM- Juv DM			0.863
			Pre DM- Adu WM			0.004
			Pre DM- Adu DM			1
			Juv WM-Juv DM			1
			Juv WM-Adu WM			1
			Juv WM-Adu DM			1
			Juv DM-Adu WM			1
			Juv DM-Adu DM			1
			Adu WM-Adu DM			0.624
**Figure S4**						
Figure S4B	Linear model per				F-statistic:	
(right)	brain area	IL	12 mice in each area	1, 10	1.824	0.207
	performance ~	PL		1, 10	1.906	0.197
	average # cells	Cg		1, 10	2.594	0.138
		OFC		1, 10	0.97	0.348
		dStr		1, 10	3.296	0.099524
		vStr		1, 10	0.2412	0.634
		CLA		1, 10	1.974	0.19

		MD		1, 10	2.449	0.149
		dHP		1, 10	2.459	0.148
		vHP		1, 10	1.854	0.203
		LEC		1, 10	2.502	0.145
		M2		1, 10	1.843	0.204
		S1		1, 10	0.1313	0.725
		V1		1, 10	1.361	0.270472
Figure S4D (right)	Linear model per brain area performance ~ average # cells				F-statistic:	
		IL	12 mice in each area	1, 10	0.06504	0.804
		PL		1, 10	3.795	0.08
		Cg		1, 10	2.779	0.12645
		OFC		1, 10	9.834	0.010584
		dStr		1, 10	0.4028	0.5399
		vStr		1, 10	0.08552	0.776
		CLA		1, 10	1.308	0.2794
		MD		1, 10	0.2037	0.661
		dHP		1, 10	0.1867	0.675
		vHP		1, 10	2.932e-06	0.999
		LEC		1, 10	0.6347	0.4442
		M2		1, 10	0.9731	0.34718
		S1		1, 10	0.6637	0.43424
		V1		1, 10	2.256	0.164

Statistics table S2. Statistics for sex effect on LME model.

Figure	Factor of sex effect	Male vs. female	Chi-Square	p-value
**Figure 1**				
Figure 1B	Age group	Entries<br>Males: 19 mice<br>Females: 16 mice	0	1
Figure 1C	Age group	Alternations (%)<br>Males: 19 mice<br>Females: 16 mice	0	1
Figure 1D	Age group	Center (%)<br>Males: 19 mice<br>Females: 16 mice	0	1
Figure 1E	Age group	Clockwise (%)<br>Males: 19 mice<br>Females: 16 mice	0	1
Figure 1F	Age group	Average speed during movement<br>Males: 19 mice<br>Females: 16 mice	0	1
Figure 1G	Age group	Total distance<br>Males: 19 mice<br>Females: 16 mice	0	1
Figure 1H	Age group	Movement (%)<br>Males: 19 mice<br>Females: 16 mice	0	1
**Figure 2**				
Figure 2C	Age group	Performance (% correct trials)<br>Males: 19 mice<br>Females: 16 mice	0	1
Figure 2D	Age group	Performance (time)<br>Males: 18 mice<br>Females: 15 mice	0	1
**Figure 3**				
Figure 3B (top)	Age group	CD trials<br>Males: 18 mice<br>Females: 18 mice	0	1
Figure 3B (middle)	Age group	CD latency<br>Males: 18 mice<br>Females: 18 mice	0	1
Figure 3B (bottom)	Age group	CD variance<br>Males: 18 mice<br>Females: 18 mice	0	1
Figure 3C (top)	Age group	CDR trials<br>Males: 18 mice	0.176	0.674

		Females: 18 mice		
Figure 3C (middle)	Age group	CDR latency<br>Males: 18 mice<br>Females: 18 mice	0.177	0.674
Figure 3C (bottom)	Age group	CDR variance<br>Males: 18 mice<br>Females: 18 mice	1.737	0.187
Figure 3D (top)	Age group	IDSS trials<br>Males: 18 mice<br>Females: 18 mice	0	1
Figure 3D (middle)	Age group	IDSS latency<br>Males: 18 mice<br>Females: 18 mice	0	1
Figure 3D (bottom)	Age group	IDSS variance<br>Males: 18 mice<br>Females: 18 mice	0	1
Figure 3E (top)	Age group	EDSS trials<br>Males: 18 mice<br>Females: 18 mice	0	1
Figure 3E (middle)	Age group	EDSS latency<br>Males: 18 mice<br>Females: 18 mice	0	1
Figure 3E (bottom)	Age group	EDSS variance<br>Males: 18 mice<br>Females: 18 mice	0	1
**Figure 4**				
Figure 4G	Age group<br>Brain area<br>Age group * Brain area	cFos expression:<br>12 mice, 230 slices (contain multiple areas), 1039 images x brain areas.<br>Males: 453 images x brain areas.<br>Females: 586 images x brain areas.	0	1
**Figure 5**				
Figure 5B (right)	Age group<br>Brain area<br>Age group * Brain area	cFos expression:<br>12 mice, 230 slices (contain multiple areas), 1039 images x brain areas.<br>Males: 184 images x brain areas.<br>Females: 906 images x brain areas.	0	1
Figure 5C (right)	Age group<br>Brain area<br>Treatment<br>Treatment * Age group * Brain area	cFos expression:<br>All mice from Figure 5B (right) as Treatment group<br>All mice from Figure 4G as Control group	0	1
Figure 5D (right)	Age group<br>Brain area	cFos expression:<br>12 mice, 230 slices (contain multiple areas), 1039 images x brain areas.	0	1

	Age group * Brain area	Males: 210 images x brain areas.<br>Females: 1063 images x brain areas.		
Figure 5E (right)	Age group<br>Brain area<br>Treatment<br>Treatment * Age group * Brain area	cFos expression:<br>All mice from Figure 5D (right) as Treatment group<br>All mice from Figure 4G as Control group	0	1
**Figure S1**				
Figure S1A	Age group	Weight 1st day<br>Males: 19 mice<br>Females: 16 mice	10.867	9.78e-04
Figure S1A	Age group	Weight change 2nd day<br>Males: 19 mice<br>Females: 16 mice	0	1
Figure S1C	Age group	Weight 1st day<br>Males: 18 mice<br>Females: 18 mice	18.737	1.50e-05
Figure S1A	Age group	Weight change 2nd day<br>Males: 19 mice<br>Females: 16 mice	0.006	0.939
